# Uncharted phytochemical frontiers: a systematic review of novel bioactive compounds from underexplored aromatic plants for cancer therapy

**DOI:** 10.3389/fonc.2026.1924878

**Published:** 2026-08-28

**Authors:** Biswabhusan Dash, Himadri Tanaya Behera, N.G. Unnoottee, Swagat Mohanty, Divaker Shukla, Sudipta Jena, Asit Ray, Pratap Chandra Panda, Sanghamitra Nayak, Ambika Sahoo

**Affiliations:** 1 Centre for Biotechnology, School of Pharmaceutical Sciences, Siksha ‘O’ Anusandhan (Deemed to be University), Bhubaneswar, Odisha, India; 2 Imgenex India Pvt. Ltd., Bhubaneswar, Odisha, India; 3 Department of Pharmacognosy and Phytochemistry, Pharmacy Academy, Faculty of Pharmacy, IFTM University, Moradabad, Uttar Pradesh, India

**Keywords:** anticancer compounds, aromatic plants, cancer therapy, drug discovery, essential oils, natural products, phytochemistry

## Abstract

**Introduction:**

Cancer continues to cause a high degree of morbidity and mortality worldwide, making it essential for new, safer, and more effective therapies to be developed. Aromatic plants provide researchers with diverse secondary metabolites that may have useful pharmacological effects. This systematic review examines several aromatic plants that were used medicinally in the past but have not been examined recently for their potential to produce new bioactive molecules with anticancer activity.

**Methods:**

The protocol for this systematic review followed PRISMA 2020, and it aimed to assess the anticancer potential of bioactive compounds obtained from underexplored aromatic plants. Peer-reviewed articles reporting phytochemical isolation, structural characterization, biological evaluation, and mechanistic investigations were systematically retrieved and analyzed. Data on plant species, bioactive compounds, identification methods, cancer models, molecular targets, pharmacological activities, and translational evidence were extracted and synthesized.

**Results:**

In this review, a variety of phytochemicals that exhibit strong anticancer effects were identified, including the following: monoterpenes, sesquiterpenes, diterpenoids, triterpenoids, flavonoids, steroid compounds and phenylpropanoids. In addition to the existing identified compounds, several new phytochemicals, named epunctanone, PCPT-1, aspiletrein A, paeoniflorigenone, 7,8-epoxynagilactone, compound 5o, and new derivatives of phorbol esters, demonstrated powerful cytotoxic activities in many cancer models. The mechanism by which these phytochemicals exert their anticancer effect has been studied, and several pathways identified including: induction of apoptosis, blockage of cell-cycle progression, modulation of oxidative stress, inhibition of angiogenesis and metastasis, as well as regulation of various critical kinase signaling pathways (e.g. PI3K/Akt/mTOR, MAPK, STAT3, EGFR, NF-κB).

**Conclusion:**

Underexplored aromatic plants are a promising source of structurally diverse bioactive compounds with encouraging anticancer activity in preclinical studies. However, there is no convincing evidence from high-quality human clinical trials that essential oils or their constituents can directly treat or cure cancer. Further rigorous preclinical and clinical studies are needed to establish their safety and therapeutic efficacy. Challenges including phytochemical standardization, limited pharmacokinetic and toxicological data, insufficient *in vitro* validation, and lack of clinical studies continue to obstruct translational progress. Future research integrating advanced phytochemical characterization, molecular oncology, AI-assisted drug discovery, and nanotechnology-based delivery systems may facilitate the development of aromatic plant-derived anticancer therapeutics.

## 1 Introduction

Cancer is considered to be a leading issue in public health care, being responsible for a substantial part of both morbidity and mortality across the globe, despite advances in early diagnosis, precision medicine, and treatment. As described in the Global Cancer Statistics 2020 report, there were approximately 19.3 million new cases and 10 million cancer deaths in the world, with the cancer burden expected to increase owing to population growth, ageing, urbanization, and lifestyle risks (1, 2). While considerable progress has been achieved in standard medical treatment modalities such as surgery, radiation, chemotherapy, targeted therapy, and immunotherapy, the efficacy of such treatments continues to be limited owing to issues such as drug resistance, tumour heterogeneity, toxicity, recurrence, and poor outcomes (3, 4). The above-mentioned challenges demonstrate the urgent need for better anticancer agents.

Traditional and natural products served as the foundation of most successful anticancer drug discoveries. Several important anticancer medications, such as paclitaxel, vincristine, vinblastine, irinotecan, topotecan, and etoposide, are derived from plant secondary metabolites, either directly or indirectly (5). The exclusive chemical diversity and wide-ranging biological activities of plant-derived compounds make them vital resources for anticancer drug discovery. Many of these compounds exert their effects by simultaneously targeting multiple cancer-related molecular pathways (6). Nearly 60% of accepted medicines for fighting cancer come from natural sources, highlighting the significant contribution of phytochemicals in anticancer drug discovery (5).

Among medicinal plants, aromatic species are a particularly valuable but relatively less explored source of bioactive compounds. They are a source of a wide range of volatile and non-volatile secondary metabolites, including monoterpenes, sesquiterpenes, diterpenoids, triterpenoids, flavonoids, phenylpropanoids, coumarins, and steroidal compounds (7, 8). Aromatic plants have traditionally been used for herbal medicine, food preservation, cosmetics and aromatherapy. They have different pharmacological activities, including antioxidant, antimicrobial, anti-inflammatory, immunomodulatory, and anticancer potential (9, 10). There is growing evidence that these phytochemicals can modulate multiple hallmarks of cancer *via* mechanisms including induction of apoptosis, cell-cycle arrest, anti-angiogenesis, inhibition of metastasis, modulation of oxidative stress, and suppression of tumour-promoting inflammation (11). In addition, many compounds obtained from aromatic plants regulate the key oncogenic signaling pathways, such as PI3K/Akt/mTOR, MAPK, STAT3, EGFR, and NF-κB, to suppress cancer cell proliferation, survival, migration, and therapeutic resistance (12–14).

Despite these favorable biological attributes, scientific inquiry has largely centred on a restricted group of widely recognized aromatic species, such as *Thymus vulgaris*, *Origanum syriacum*, *Mentha* spp., and *Cinnamomum zeylanicum* (13, 15–18). However, many aromatic plant species found in the biodiversity-rich regions of Asia, Africa, South America, and the Mediterranean remain largely unexplored. Although many of these plants have a long history of use in traditional medicine and are known to produce structurally diverse metabolites, their potential as sources of anticancer compounds has yet to be thoroughly investigated (19). Advances in analytical techniques such as separation by chromatography, metabolomics, high-resolution mass spectrometry, nuclear magnetic resonance spectroscopy and bioassay-guided fractionation have greatly facilitated the identification of new phytochemicals from these neglected plants (20–22).

Several recent preclinical studies suggest that these underexplored aromatic plants are promising sources of bioactive compounds for anticancer drug discovery. However, the current evidence is largely based on *in vitro* and animal studies, and robust clinical evidence in humans remains lacking. Many novel compounds, including epunctanone from *Garcinia epunctata*, PCPT-1 from *Leptopus lolonum*, aspiletrein-A from *Aspidistra letreae*, paeoniflorigenone from *Paeonia suffruticosa*, and Type B nagilactones from *Podocarpus nagi* possess strong antiproliferative abilities through their actions that induce apoptosis, inhibit metastasis, and modulate PI3K/Akt and MAPK pathways (23–27). Further the essential oils extracted from *Hedychium spicatum, Aloysia citriodora, Chrysopogon zizanioides, Boswellia serrata, Spilanthes paniculata*, and *Pallenis spinosa* possess significant antiproliferative, pro-apoptotic, antiangiogenic, and anti-metastatic properties in several experimental models of cancers (20, 21, 28–31).

However, the clinical translation of aromatic plant-derived compounds remains limited due to challenges such as variability in phytochemical composition, lack of standardized extraction protocols, insufficient pharmacokinetic and toxicological evaluation, and limited *in vitro* and clinical validation (6). Although the number of studies investigating these plants has increased considerably, the available evidence remains fragmented and has not been comprehensively synthesized with a focus on underexplored aromatic species and their novel bioactive constituents.

Therefore, the present systematic review aims to provide an overview of new bioactive molecules from aromatic plants that have been underexplored, and growing preclinical evidence highlights the potential of these underexplored aromatic plants as sources of lead compounds for anticancer drug discovery and future therapeutic development. However, this potential remains unconfirmed in humans, as the current evidence is derived predominantly from *in vitro* and animal studies. This review aims to present the available evidence in the literature regarding aromatic plant diversity, their phytochemistry, anticancer properties, underlying molecular mechanisms, signaling pathways, potential for translation into treatments, and possible future treatment modalities. This review focuses on lead compounds and the research gaps currently existing in the field. To systematically appraise the available evidence for these observations, a comprehensive systematic review was performed following a predesigned methodology.

## 2 Methodology

### 2.1 Study design

This systematic review aimed to find, assess, and combine the available evidence on new bioactive compounds from lesser-known aromatic plants and their possible uses in cancer treatment. The review was planned and described following the PRISMA 2020 guidelines (32). The goal was to investigate phytochemicals obtained from aromatic plants that exhibit anticancer properties, particularly with respect to their structural diversity, biological activity, mechanisms, and possible applications.

### 2.2 Literature search strategy

A comprehensive literature search was conducted independently in PubMed, Scopus, and ScienceDirect to identify studies published between January 2000 and March 2026 investigating aromatic plants, essential oils, phytochemicals, natural products, and their anticancer activities. Database-specific search strategies incorporated relevant keywords, Boolean operators, and Medical Subject Headings (MeSH), including terms such as underexplored aromatic plants, essential oils, bioactive compounds, phytochemicals, anticancer, cytotoxic, antitumour, and cancer therapy. The complete search strategies for each database are provided in **Supplementary Table 1**. The final electronic search was performed on 31 March 2026, followed by manual screening of the reference lists of eligible articles to identify additional relevant studies. All retrieved records were imported into Rayyan AI for duplicate removal and independent title, abstract, and full-text screening.

### 2.3 Eligibility criteria

The screened articles based on the inclusion and exclusion criteria consisted of original peer-reviewed articles on aromatic plant species with bioactive compounds along with anticancer and antiproliferative activity experimentally verified by various methods. We included studies using *in vitro* and/or *in vivo* cancer models and/or studies describing molecular mechanisms or signaling pathways of the anticancer activities. Review articles, editorials, conference abstracts, book chapters, patents, non-English publications, and studies lacking phytochemical characterization or sufficient biological validation were excluded from the analysis.

### 2.4 Study selection

**Figure 1:**
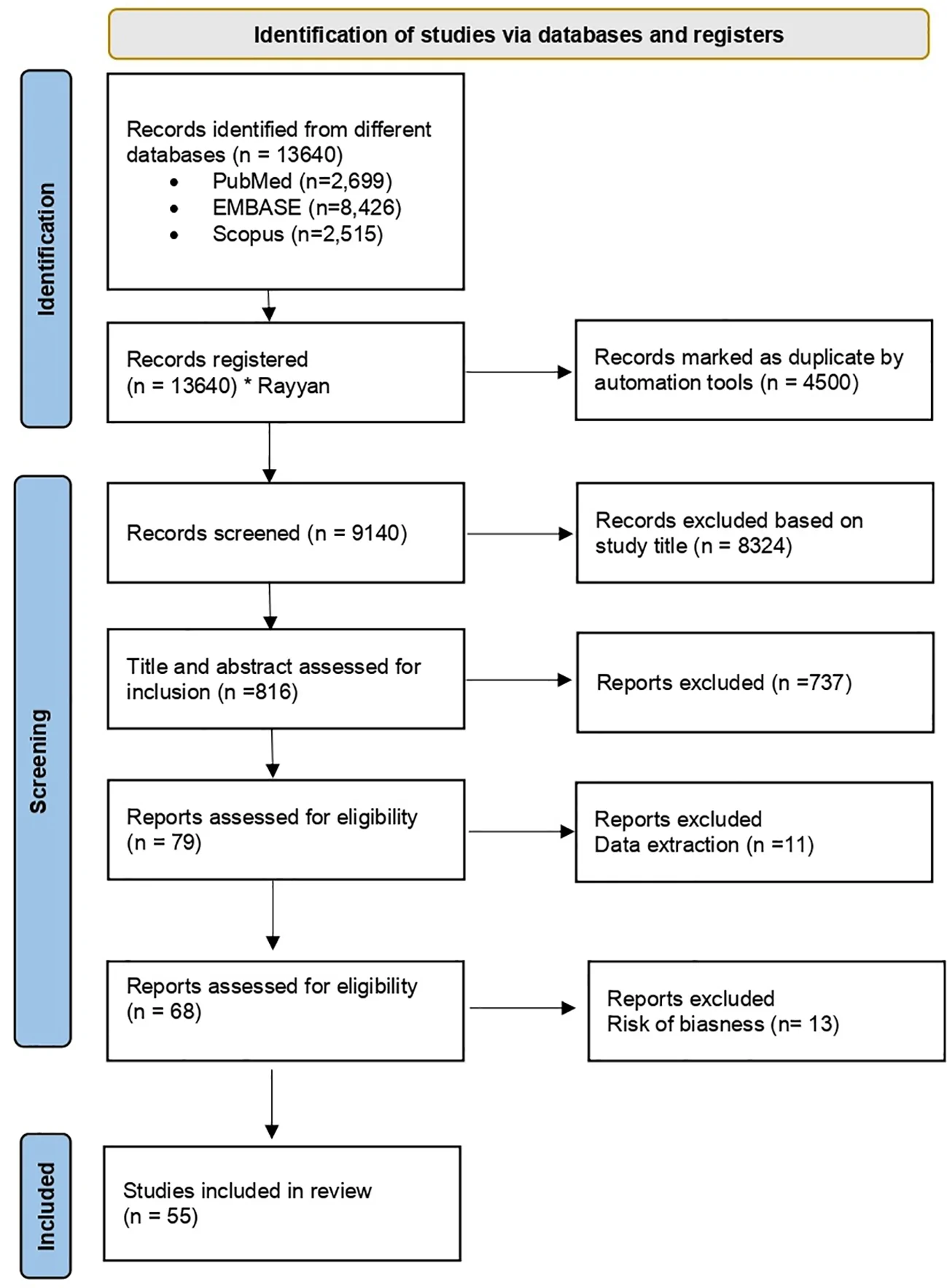
PRISMA 2020 flow diagram.

The process of study selection occurred in several stages. Duplicates were removed, and titles and abstracts were screened for relevance to the review objectives. Those studies that were potentially eligible were then evaluated in full text. Any disagreements during study selection were resolved through discussion and consensus of the reviewers. The literature search yielded 13,640 records, of which 4,500 duplicate entries were removed. The remaining 9,140 records underwent title and abstract screening, leading to the exclusion of 8,324 irrelevant studies. A total of 816 full-text articles were assessed for eligibility. Out of these, 737 studies were excluded because they did not meet the inclusion criteria. The remaining 79 studies were included for data extraction. During this process, 11 studies were discarded due to incomplete phytochemical or biological information. The remaining 68 studies were subjected to methodological quality assessment, and 13 studies identified as having a high risk of bias were excluded. Ultimately, 55 studies were included in the final qualitative synthesis. The study selection process is shown in the PRISMA 2020 flow diagram (**Figure 1**).

### 2.5 Data extraction

The process of data extraction was done through an established extraction framework that was designed for the review. The information that was obtained from the study comprised plant name, botanical family, geographic source, part of the plant utilized, process of extraction and isolation, methodology used to identify the phytochemicals, chemical classes of phytochemicals, novelty of the phytochemicals, cancer models used, experiment design, parameters of biological activity, mechanisms of action of the phytochemicals, signaling pathways involved, and information regarding *in vitro* efficacy, toxicity, and translational potential.

### 2.6 Risk of bias assessment

To assess the methodological quality of the included studies, a customized 20-item risk-of-bias checklist was developed based on key quality domains in phytochemical and preclinical anticancer research. Although it provided a structured framework for quality assessment, the checklist has not been formally validated and should therefore be considered an author-developed tool. Important aspects taken into account during assessment were plant authentication, extraction procedures, phytochemical characterization, experimental controls, biological validation, mechanistic investigations, statistical analyses, reproducibility and transparency of reporting. Each criterion was scored as either 1 (criterion fulfilled) or 0 (criterion not fulfilled or inadequately reported). Studies were subsequently categorized as low, moderate, or high risk of bias based on their cumulative scores. Only studies with acceptable methodological quality were retained for the final synthesis.

### 2.7 Data synthesis and analysis

Considering the large heterogenecity between the included studies concerning the plant species, phytochemicals used, methods of experimentation and measurements of outcomes, quantitative meta-analysis was not thought appropriate. Therefore, we used a narrative synthesis approach. The results were categorized according to aromatic plant diversity, phytochemical composition, anticancer activities, molecular mechanisms, signaling pathways, translational potential and future therapeutic opportunities. The comparisons pointed out the potentially valuable lead candidates, novel molecular targets, as well as essential research limitations that can suggest the directions of future investigation in aromatic plant-based cancer drugs.

## 3 Results and discussion

### 3.1 Study selection and characteristics of included studies

From the electronic database, a total of 13,640 literature sources were identified through the systematic search. However, after removing 4,500 duplicate studies using the Rayyan AI tool, a total of 9,140 unique literature sources were identified, of which 8,324 were excluded for non-compliance with the predefined inclusion criteria. This led to a total of 816 full-text eligible articles. Subsequently, 79 studies underwent detailed data extraction; 11 were excluded due to incomplete phytochemical or biological information. The remaining 68 studies underwent a methodological quality and risk-of-bias assessment. After the quality assessment, 13 studies were excluded. Consequently, 55 studies were included in the final qualitative synthesis (**Figure 1**).

The included studies provide substantial evidence supporting the anticancer potential of phytochemicals derived from underexplored aromatic plants. These investigations encompassed species belonging to diverse botanical families, including Lamiaceae, Asteraceae, Apiaceae, Rutaceae, Lauraceae, Zingiberaceae, Burseraceae, Euphorbiaceae, Podocarpaceae, Clusiaceae, Asparagaceae, and Paeoniaceae (**Table 1**). Most studies were published between 2016 and 2026, indicating growing scientific interest in aromatic plant-based cancer therapeutics (23, 24, 27).

Most of the studies employed *in vitro* cancer models and a smaller percentage included *in vitro* validation using xenograft, orthotopic, syngeneic or chemically induced tumour models. Breast cancer, colorectal cancer, lung cancer, gastric cancer, liver cancer, prostate cancer, ovarian cancer, oral cancer, and glioblastoma were among the most frequently investigated malignancies (12, 13, 21, 57).

The review revealed a strong emphasis on essential oil-based studies. In particular, essential oils enriched with monoterpenes and sesquiterpenes were the most extensively investigated phytochemical preparations. However, recent studies increasingly employed bioassay-guided fractionation, chromatographic purification, and advanced spectroscopic techniques to isolate individual compounds with defined chemical structures and biological targets (20, 29, 59). The use of phytochemical characterization combined with biological authentication has greatly improved the identification of promising lead molecules from underexplored aromatic plants. The diversity of the included aromatic plants provides the foundation for understanding the structural diversity of their bioactive constituents and their subsequent biological activities.

### 3.2 Diversity of underexplored aromatic plants and their bioactive compounds

The research articles included in this systematic review are taxonomically, phytochemically, and pharmacologically varied, with diverse aromatic plants under-researched and underexplored for their anticancer properties. These species represent a valuable but largely untapped source of bioactive metabolites capable of targeting multiple hallmarks of cancer. The information contained in **Tables 1** and **2** indicates that the plants, native to various families of a broad spectrum, when found to synthesize compounds that vary in structure may have important antitumour activities. This may suggest that these plants could be a potential source of new anticancer drugs (21, 28, 30, 71).

#### 3.2.1 Aromatic plant species identified

Overall, 55 studies examining diverse aromatic plant species were included in the qualitative synthesis. The investigated plants belonged predominantly to the families Lamiaceae, Asteraceae, Apiaceae, Rutaceae, Lauraceae, Zingiberaceae, Burseraceae, Euphorbiaceae, Podocarpaceae, Clusiaceae, Asparagaceae, and Paeoniaceae. Species such as *Hedychium spicatum, Aegle marmelos, Aloysia citriodora, Plectranthus amboinicus, Plectranthus cylindraceus, Meriandra dianthera, Origanum onites, Thymus vulgaris, Salvia pisidica, Boswellia serrata, Chrysopogon zizanioides, Spilanthes paniculata, Heracleum persicum, Tanacetum sinaicum, Podocarpus nagi, Gnidia involucrata, Aspidistra letreae*, and *Paeonia suffruticosa* emerged as important sources of anticancer phytochemicals.

The geographical distribution of these species highlighted the significance of biodiversity-rich regions in natural product discovery. Several plants originated from Asia, the Mediterranean basin, Africa, and the Middle East, areas recognized for their rich ethnomedicinal traditions and unique aromatic flora. Moreover, for most of the species investigated in this study, less research work concerning cancer activity has been conducted, highlighting value of investigation for less investigated aromatic plant resources.

#### 3.2.2 Plant parts and extraction methods

The inclusion of different plant parts was observed throughout the studies, which is due to the localization of the secondary metabolites in aromatic plants. Leaves, flowers, aerial parts, bark, rhizomes, fruits, seeds, stems, and roots were the most commonly investigated materials. Leaves and aerial parts represented the predominant sources of essential oils, while roots, rhizomes, bark, and seeds frequently yielded non-volatile phytochemicals with significant biological activities.

Hydrodistillation and steam distillation were the most commonly used methods for extracting essential oils (72). These techniques efficiently recovered volatile monoterpenes and sesquiterpenes, which are responsible for many of the reported anticancer activities (73). In contrast, non-volatile phytochemicals were typically extracted using solvents such as methanol, ethanol, ethyl acetate, chloroform, hexane, or hydroalcoholic mixtures (74). Several studies further employed bioassay-guided fractionation and chromatographic purification techniques, including column chromatography, preparative high-performance liquid chromatography (HPLC), and flash chromatography, to isolate individual compounds for biological evaluation (75, 76).

The rising popularity of bioassay-guided methodology is due to the transformation from crude extract screening to the discovery of purified, isolated bioactive molecules which have well-known pharmacological activities, to develop potent antitumour agents from natural products.

#### 3.2.3 Chemical diversity of phytochemicals

**Figure 2:**
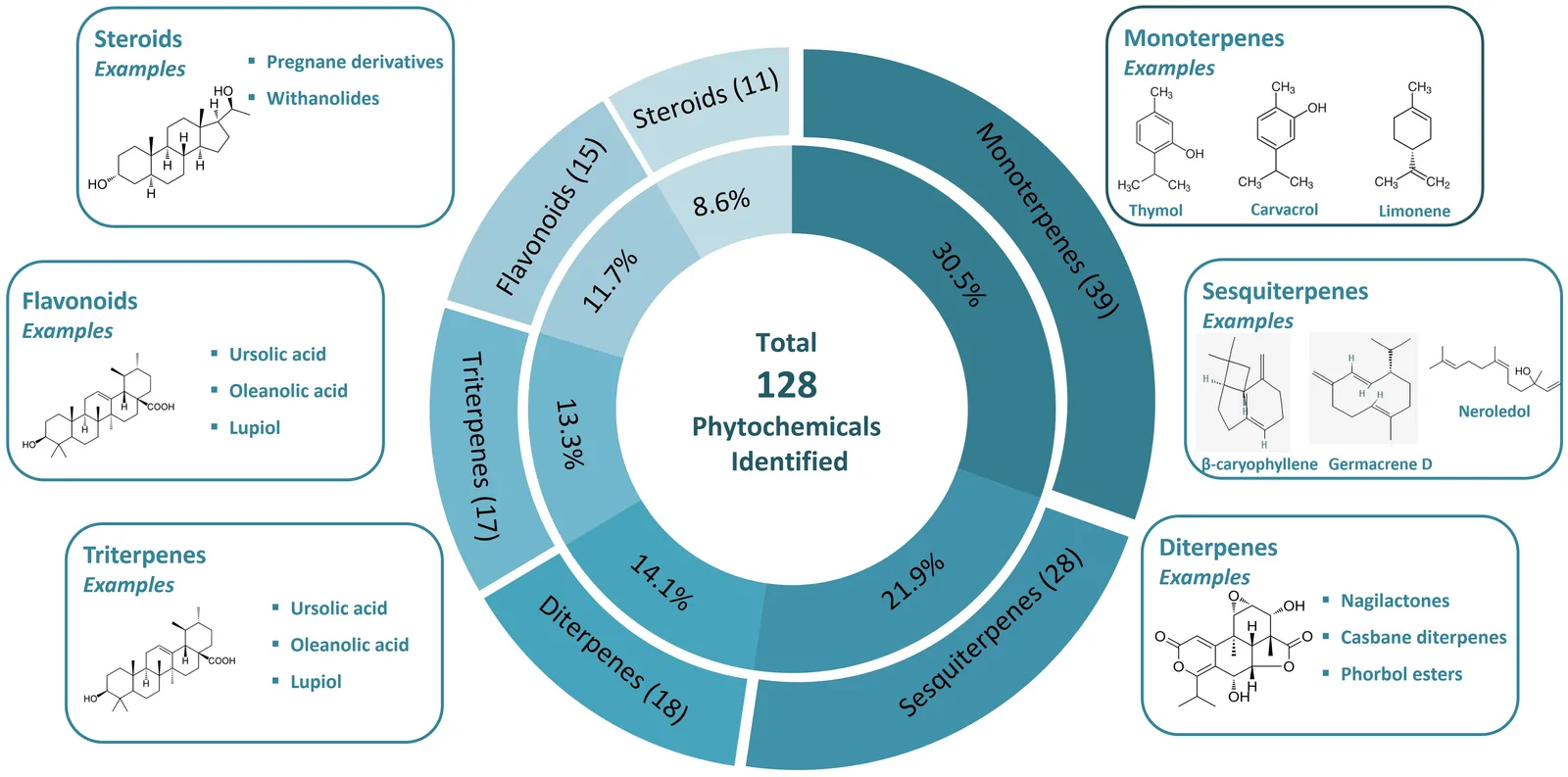
Chemical diversity of Identified anticancer phytochemicals.

The included studies revealed extensive phytochemical diversity, with a wide range of secondary metabolites identified, including monoterpenes, sesquiterpenes, diterpenoids, triterpenoids, flavonoids, coumarins, lignans, phenylpropanoids, steroidal compounds, and other specialized metabolites. Among these, terpenoids represented the most abundant and biologically active class of compounds (**Figure 2**).

Frequently reported monoterpenes included thymol, carvacrol, *α*-pinene, limonene, citral, borneol, and bornyl acetate, many of which exhibited potent antiproliferative and pro-apoptotic activities (13, 14, 49). Sesquiterpenes such as *β*-caryophyllene, germacrene D, nerolidol, khusimol, δ-cadinene, and *α*-vetivone also demonstrated significant anticancer properties. In addition, several studies identified diterpenoids, triterpenoids, and steroidal metabolites with superior potency compared to many common essential oil constituents (36, 61). A high degree of chemical variability has been described here, and this demonstrates a rich biosynthetic capability of the plant species and shows that they can produce compounds of diverse structures and a variety of possible modes of interaction with various cellular targets which participate in the cancer process.

#### 3.2.4 Novel compounds discovered from underexplored species

One of the most important findings of this review is the discovery of several novel and underexplored compounds that exhibit promising anticancer activity. Epunctanone isolated from *Garcinia epunctata* demonstrated potent cytotoxic activity against multidrug-resistant cancer cells through induction of apoptosis and ferroptosis (23). Similarly, PCPT-1, a novel pentacyclic triterpenoid isolated from *Leptopus lolonum*, exhibited significant antiproliferative activity against hepatocellular carcinoma cells (24). Additional noteworthy discoveries included aspiletrein A from *Aspidistra letreae*, paeoniflorigenone from *Paeonia suffruticosa*, and several novel type-B nagilactones isolated from *Podocarpus nagi* (25–27). Among these, 7*α*,8*α*-epoxynagilactone demonstrated exceptional potency against epidermoid carcinoma cells. Discovery of highly active phorbol ester and pregnane steroid derivatives of *Gnidia involucrata* and *Acokanthera schimperi* strengthens the evidence for targeting understudied aromatic plant species to develop anticancer scaffolds.

These findings highlight the potential of biodiversity-driven phytochemical research to identify structurally unique compounds with mechanisms of action that differ from those of current anticancer therapies.

#### 3.2.5 Phytochemical characterization techniques

Improvements in analysis and spectroscopic technologies were essential to detect bioactive compounds in plants. Gas chromatography-mass spectrometry (GC-MS) was the most commonly employed technique for profiling essential oil constituents and volatile metabolites. Several studies further utilized comprehensive two-dimensional gas chromatography coupled with time-of-flight mass spectrometry (GC×GC-TOFMS) for higher-resolution characterization of complex mixtures in essential oils.

Non-volatile compounds were routinely separated and identified using liquid chromatography-mass spectrometry (LC-MS/MS), ultra-performance liquid chromatography-high-resolution mass spectrometry (UPLC-HRMS), and high-performance liquid chromatography (HPLC). Structural elucidation was primarily achieved through one-dimensional and two-dimensional nuclear magnetic resonance (1D and 2D NMR) spectroscopy, often supported by infrared spectroscopy and high-resolution mass spectrometry.

The coupling of sophisticated analytical technologies and bioassay-guided fractionation accelerated the isolation of new natural products and improved the structural elucidation process. These methodological advances have played a pivotal role in expanding our understanding of the chemical diversity and therapeutic potential of underexplored aromatic plants (20, 21, 30).

Based on the evidence presented in **Tables 1** and **2**, under-exploited aromatic plants constitute an important reservoir of bioactive compounds exhibiting a wide range of biological activities. More research on these plants through more sophisticated methods of phytochemical investigation will most likely lead to the discovery of new compounds of medicinal value, especially anticancer drugs. After characterizing the botanical diversity and phytochemical composition of these species, the next section summarizes their experimentally reported anticancer activities across different cancer models.

**Table 1:** Characteristics of Included Studies.

Aromatic Plant	Family	Plant Part	Preparation	Cancer Model	Study Design	Main Finding	Reference
*Plectranthus amboinicus*	Lamiaceae	Leaves	Essential oil	H1299	*In vitro*	IC50 = 11 µg/mL	Abdalla et al., (33)
*Origanum vulgare* ssp. *hirtum*	Lamiaceae	Aerial parts	Essential oil	CT26/BALB-c	*In vivo*	44% tumor reduction	Aindelis et al., (34)
*Trachyspermum ammi*	Apiaceae	Seeds	TSEO-PNP	HT-29	*In vitro*	Apoptosis induction	Almnhawy et al., (35)
*Jasonia glutinosa*	Asteraceae	Aerial parts	Essential oil	MCF-7, HepG2	*In vitro*	Selective cytotoxicity	Baraich et al., (36)
*Mentha aquatica* var. Kenting Water Mint	Lamiaceae	Whole plant	Essential oil	Skin carcinoma	*In vitro/In vivo*	Chemopreventive activity	Chang et al., (37)
*Taiwania cryptomerioides*	Cupressaceae	Bark	EO fraction	HepG2	*In vitro*	Active diterpenoid isolated	Chen et al., (38)
*Croton matourensis*	Euphorbiaceae	Leaves	Essential oil	Sarcoma 180	*In vitro/In vivo*	Tumor inhibition	Lima et al., (39)
*Amomum villosum*	Zingiberaceae	Fruits	Aromatic oil	Gastric cancer	*In vitro/In silico*	HMOX1-mediated effect	Deng et al., (40)
*Pinus sylvestris*	Pinaceae	Needles	Essential oil	U-87MG, SH-SY5Y	*In vitro*	ROS-mediated apoptosis	Dervişoğlu, (41)
*Rosmarinus officinalis*	Lamiaceae	Leaves	Essential oil	HCT116	*In vitro*	Significant cytotoxicity	Dolghi et al., (42)
*Hedychium spicatum*	Zingiberaceae	Rhizome	Essential oil	PC-3, HCT-116, A549	*In vitro*	Selective cytotoxicity and apoptosis induction	Ray et al., (21)
*Nigella sativa*	Ranunculaceae	Seeds	Thymoquinone	Multiple cancer models	*In vitro/In silico*	Potent anticancer activity	Ravi et al., (43)
Diosmetin-containing plants	Various	Various	Diosmetin	EAC, liver and lung cancer	*In vitro/In vivo*	ROS-mediated tumor suppression	Raza et al., (44)
*Chrysopogon zizanioides*	Poaceae	Roots	Essential oil	HCT-116, A549	*In vitro/In silico*	Antiproliferative activity	Karousa et al., (20)
*Thymus vulgaris*	Lamiaceae	Aerial parts	Extract	Breast carcinoma	*In vitro/In vivo*	Anticancer and chemopreventive effects	Kubatka et al., (13)
*Cinnamomum zeylanicum*	Lauraceae	Bark	Extract	Breast carcinoma	*In vitro/In vivo*	Tumor suppression and apoptosis	Kubatka et al., (14)
*Polyalthia longifolia*	Annonaceae	Whole plant	Clerodane diterpene derivatives	Colon cancer	*In vitro/In vivo*	Compound 5o showed potent antitumor activity	Kumar et al., (45)
*Aloysia citriodora*	Verbenaceae	Leaves	Essential oil	Melanoma	*In vitro*	Strong cytotoxic activity	Al-Maharik et al., (29)
*Garcinia epunctata* / *Ptycholobium contortum*	Clusiaceae/Fabaceae	Various	Isolated compounds	MDR cancer cells	*In vitro*	Activity against resistant cancers	Mbaveng et al., (23)
*Plectranthus* spp.	Lamiaceae	Leaves	Essential oil	HeLa, HepG2, HT-29	*In vitro*	Strong cytotoxicity	Mothana et al., (46)
*Meriandra dianthera*	Lamiaceae	Aerial parts	Essential oil	MCF-7, HepG2	*In vitro*	Apoptosis induction	Mothana et al., (47)
*Aspidistra letreae*	Asparagaceae	Whole plant	Aspiletrein A	NSCLC	*In vitro*	Anti-metastatic activity	Nguyen et al., (25)
*Paeonia suffruticosa*	Paeoniaceae	Root bark	Paeoniflorigenone	HNSCC	*In vitro*	Multiple programmed cell death pathways	Park et al., (26)
*Leptopus lolonum*	Euphorbiaceae	Whole plant	PCPTs	HepG2	*In vitro*	MAPK/Akt-mediated apoptosis	Qi et al., (24)
*Plectranthus ecklonii*	Lamiaceae	Leaves	Parvifloron D	TNBC	*In vitro*	Anti-migratory activity	Saraiva et al., (48)
*Pistacia atlantica*	Anacardiaceae	Oleoresin	Essential oil	AGS, MKN-45	*In vitro*	Bax/Bcl2-mediated apoptosis	Saeedi et al., (49)
–	–	–	Thymol, Carvacrol, Eugenol, Geraniol, 1,8- Cineol, borneol, 2-Undecanone	MCF-7, T24	*In vitro/In silico*	Caspase-dependent apoptosis	Saini et al., (50)
*Pallenis spinosa*	Asteraceae	Flowers/Leaves	Essential oil	Breast cancer	*In vitro*	Cell-cycle arrest and apoptosis	Saleh et al., (28)
*Zataria multiflora*	Lamiaceae	Aerial parts	Nanoemulsified EO	Drug-resistant breast cancer	*In vitro*	Enhanced cytotoxicity	Salehi et al., (10)
*Toona sinensis*	Meliaceae	Leaves	Essential oil	SGC-7901, HepG2, HT29	*In vitro*	Induced apoptosis through Bax↑, Caspase-3↑, Bcl-2↓	Wu et al., (51)
*Carpesium abrotanoides*	Asteraceae	Whole plant	Sesquiterpene lactones	A549, HepG2, HCT116, MDA-MB-231, CNE2	*In vitro*	Compound 6 induced G2/M arrest and ROS accumulation	Yang et al., (52)
*Boswellia serrata*	Burseraceae	Gum resin	Essential oil	MDA-MB-231	*In vitro*	Apoptosis, ROS generation, synergism with doxorubicin	Zhang et al., (31)
*Podocarpus nagi*	Podocarpaceae	Twigs	Nagilactones	A431 and other cancer cells	*In vitro*	Compound 1 (IC50 0.208 μM) induced apoptosis and G1 arrest	Zheng et al., (27)
*Cinnamomum zeylanicum*	Lauraceae	Bark	Essential oil	HCT116	*In vitro*	Selective antitumor activity and G1 arrest	Živković et al., (53)
*Spilanthes paniculata*	Asteraceae	Flower and Leaf	Essential oil	SCC9	*In vitro/In silico*	Flower EO more potent (IC50 113.95 μg/mL)	Satapathy et al., (30)
*Salvia pisidica*	Lamiaceae	Aerial parts	Essential oil	MCF-7, Caco-2, HepG2, LnCap	*In vitro*	Triggered intrinsic and extrinsic apoptosis	Semiz et al., (54)
*Tanacetum sinaicum*	Asteraceae	Aerial parts	Essential oil	HeLa, MCF-7	*In vitro*	Antiproliferative and anti-metastatic activity	Sheashea et al., (55)
*Ocimum tenuiflorum*	Lamiaceae	Aerial parts	Essential oil	A549	*In vitro/In silico*	IC50 38.28 μg/mL; EGFR inhibition	Singh et al., (56)
*Coriandrum sativum*	Apiaceae	Aerial parts	Essential oil	A549	*In vitro/In silico*	IC50 74.53 μg/mL	Singh et al., (56)
*Origanum onites*	Lamiaceae	Aerial parts	Essential oil	HT-29, CT26	*In vitro/In vivo*	Antitumor activity after oral administration	Spyridopoulou et al., (57)
*Artemisia turanica*	Asteraceae	Aerial parts	Essential oil	HeLa	*In vitro*	IC50 17.67 μg/mL against cancer cells	Taherkhani, (58)
*Acokanthera schimperi*	Apocynaceae	Whole plant	Pregnane steroid	A427, Dan-G, MCF-7, SiSo	*In vitro*	GI50 2.3–4.7 μM; G2/M arrest	Tesfaye et al., (59)
*Gnidia involucrata*	Thymelaeaceae	Whole plant	Phorbol ester	Dan-G	*In vitro*	GI50 0.3 nM; induced apoptosis	Tesfaye et al., (59)
*Seseli bocconei*	Apiaceae	Stems	Essential oil	HCT116	*In vitro*	97% cell death at 250 μg/mL	Vaglica et al., (60)
*Seseli tortuosum* subsp. *maritimum*	Apiaceae	Stems	Essential oil	HCT116	*In vitro*	82% reduction in viability	Vaglica et al., (60)
*Decatropis bicolor*	Rutaceae	Essential oil constituent	δ-Cadinene	MDA-MB-231	*In vitro/In silico*	Selective anti-invasive activity	Reyes-Vidal et al., (61)
*Cotinus coggygria*	Anacardiaceae	Leaves	Total flavonoids/Myricetin	Glioblastoma	*In vitro/In vivo*	PI3K/Akt and ERK inhibition	Wang et al., (12)

**Table 2:** Novel Bioactive Compounds from Underexplored Aromatic Plants and Their Anticancer Activities

Plant Species	Bioactive Compound(s)	Chemical Class	Identification Method	Novelty Status	Cancer Model	IC50 (µM or µg/mL)	Key Activity	Reference
*Myrtus communis*	α-Pinene, 1,8-Cineole, Limonene	Monoterpenes	GC-MS	Underexplored aromatic species from Morocco	P815	12.5 µg/mL	Cytotoxic activity	Harassi et al., (62)
*Tinospora cordifolia*	Glucobrassicin	Indole glucosinolate	GC-MS, Docking	Novel TOP2A-targeted phytochemical	MCF-7, MDA-MB-231	NR	ROS-mediated apoptosis	Muhasina et al., (63)
*Artemisia serotina*	Camphor, Borneol derivatives	Monoterpenes	GC-MS	First bioactivity report from Kazakhstan population	Multiple	NR	Cytotoxic activity	Kadyrbay et al., (64)
*Plectranthus amboinicus*	Thymol	Monoterpene phenol	GC-MS	First comparative anticancer evaluation	H1299	11 µg/mL	Strong antiproliferative activity	Abdalla et al., (33)
*Origanum vulgare* ssp. *hirtum*	Carvacrol	Monoterpene phenol	GC-MS	First preclinical colon cancer validation	CT26	NR	44% tumor suppression	Aindelis et al., (34)
*Trachyspermum ammi*	Thymol-rich EO	Monoterpene phenol	GC-MS	Nanoencapsulation-enhanced activity	HT-29	~84 µg/mL	Apoptosis induction	Almnhawy et al., (35)
*Jasonia glutinosa*	Nerolidol	Sesquiterpene alcohol	GC-MS	First comprehensive anticancer study	MCF-7	15.43 µg/mL	Selective cytotoxicity	Baraich et al., (36)
*Mentha aquatica* var. Kenting	Menthone derivatives	Monoterpenes	GC-MS	First chemoprevention report	PDV cells	NR	G2/M arrest	Chang et al., (37)
*Taiwania cryptomerioides*	6,7-Dehydroroyleanone	Diterpenoid quinone	Bioassay-guided isolation, NMR	Novel active constituent	HepG2	10.28 µM	Apoptosis induction	Chen et al., (38)
*Croton matourensis*	β-Caryophyllene, Germacrene D	Sesquiterpenes	GC-MS, GC-FID	Underexplored Amazonian species	Sarcoma 180	NR	Antitumor activity	Lima et al., (39)
*Amomum villosum*	Bornyl acetate	Monoterpene ester	GC-MS	Mechanistically validated HMOX1 inhibitor	AGS, HGC27	NR	Anti-gastric cancer activity	Deng et al., (40)
*Pinus sylvestris*	α-Pinene, β-Pinene	Monoterpenes	GC-MS	First neuro-oncology evaluation	U-87MG	47.93 µg/100 µL	ROS-mediated apoptosis	Dervişoğlu, (41)
*Rosmarinus officinalis*	Eucalyptol, α-Pinene, Camphor	Monoterpenes	GC-MS	Colorectal cancer evaluation	HCT116	~83 µg/mL	Significant cytotoxicity	Dolghi et al., (42)
*Myrciaria tenella*	α-Pinene, Caryophyllene oxide	Monoterpenes/Sesquiterpenes	GC-MS	First biological activity report	SCC-25	~20–40 µg/mL	Oral cancer cytotoxicity	Ferreira et al., (65)
*Heracleum persicum*	Hexyl butyrate	Aliphatic ester	GC-MS	First ovarian cancer evaluation	OVCAR-3	12.08 µg/mL	Cytotoxic activity	Ghavam, (66)
*Decatropis bicolor*	β-Terpineol, δ-Cadinene	Monoterpene/Sesquiterpene	GC-MS	Ethnomedicinal validation	MDA-MB-231	53.81 µg/mL	Apoptosis induction	Estanislao Gómez et al., (67)
*Cedrus atlantica*	β-Himachalene	Sesquiterpene	GC-MS	First docking-guided evaluation	A375, HT-29	NR	Mitochondrial apoptosis	Gruin et al., (68)
*Teucrium ramosissimum*	Terpenoid phytocomplex	Mixed terpenoids	FTIR, GC-MS	Novel TRAIL sensitizer	HCT-116	NR	JAK/STAT inhibition	Guesmi et al., (69)
*Lantana camara*	Flower-specific volatiles	Mixed terpenoids	GC-MS	First flower-color comparative study	MCF-7, MDA-MB-231	NR	Selective antiproliferative activity	El Hajj et al., (70)
*Garcinia epunctata*	Epunctanone	Benzophenone derivative	Column chromatography, NMR, MS	Novel benzophenone active against MDR cancer cells	HCT116, HepG2, CCRF-CEM	4.84–13.12 µM	Apoptosis, ferroptosis, ROS generation	Mbaveng et al., (23)
*Ptycholobium contortum*	Mundulea lactone, Seputhecarpan A, Seputheisoflavone	Isoflavonoids	Column chromatography, NMR, MS	First report against multidrug-resistant cancer cells	Leukemia, Breast, Colon cancer	8.84–68.66 µM	Cytotoxicity against resistant cancer cells	Mbaveng et al., (23)
*Plectranthus cylindraceus*	Maaliol	Oxygenated sesquiterpene	GC-FID, GC-MS	Underexplored Saudi aromatic plant	HeLa, HepG2, HT-29	3.8–7.5 µg/mL	Strong cytotoxicity	Mothana et al., (46)
*Plectranthus barbatus*	α-Pinene	Monoterpene	GC-FID, GC-MS	First anticancer evaluation of Saudi chemotype	HeLa, HepG2, HT-29	3.8–7.5 µg/mL	Cytotoxic activity	Mothana et al., (46)
*Meriandra dianthera*	Camphor, 1,8-Cineole	Oxygenated monoterpenes	GC, GC-MS	First anticancer assessment from Saudi Arabia	MCF-7, HepG2, Hela	83.6–91.2 µg/mL	Apoptosis induction	Mothana et al., (47)
*Aspidistra letreae*	Aspiletrein A	Steroidal saponin	Isolation, NMR, HRMS	Novel endemic Vietnamese metabolite	H460, H23, A549	Not reported	Anti-metastatic via Akt inhibition	Nguyen et al., (25)
*Aegle marmelos*	β-Caryophyllene, α-Humulene	Sesquiterpenes	GC-MS	Nepalese sesquiterpene-rich chemotype	PSN-1, LoVo, H157, 2008	2.3–6.7 µg/mL	Potent antiproliferative activity	Pant et al., (71)
*Paeonia suffruticosa*	Paeoniflorigenone	Monoterpene derivative	Isolation, HPLC, NMR	First HNSCC mechanistic evaluation	YD-10B	Not reported	Apoptosis, autophagy and necroptosis regulation	Park et al., (26)
*Leptopus lolonum*	PCPT-1	Pentacyclic triterpenoid	Isolation, HR-ESI-MS, 1D & 2D NMR	Newly identified natural compound	HepG2	11.87 µM	MAPK/Akt-mediated apoptosis	Qi et al., (24)
*Nigella sativa*	Thymoquinone	Monoterpene quinone	HPTLC, Molecular docking	Bioactive constituent validated against leukemia	K562	Significant cytotoxicity reported	Antileukemic activity	Ravi et al., (43)
*Hedychium spicatum*	β-Pinene, Eucalyptol, Sabinene	Monoterpenes	GC-TOFMS, GC×GC-TOFMS	140 compounds reported for first time	PC-3, HCT-116, A549	Selective for PC-3	ROS-mediated apoptosis	Ray et al., (21)
Citrus fruits	Diosmetin	Flavonoid	Isolation, HPLC	First EAC mechanistic study	EAC model	Not reported	ROS-mediated antitumor effect	Raza et al., (44)
*Pistacia atlantica*	α-Pinene	Monoterpene	GC-MS	First gastric cancer evaluation	AGS, MKN-45	1.94×10⁻³% ; 2.8×10⁻³%	Bax/Bcl2 mediated apoptosis	Saeedi et al., (49)
–	Thymol	Phenolic monoterpene	GC-MS, Molecular docking	Comparative EO constituent study	MCF-7, T24	24–34 µg/mL	Caspase-dependent apoptosis	Saini et al., (50)
–	Carvacrol	Phenolic monoterpene	GC-MS, Molecular docking	Comparative EO constituent study	MCF-7, T24	24–34 µg/mL	Caspase-dependent apoptosis	Saini et al., (50)
*Pallenis spinosa* Flower	Sesquiterpene-rich EO	Sesquiterpenes	GC-MS, SPME-GC-MS	First detailed breast cancer mechanistic study	MCF-7, MDA-MB-231	0.21–0.25 µg/mL	Apoptosis and G0/G1 arrest	Saleh et al., (28)
*Zataria multiflora*	Thymol, Carvacrol	Monoterpene phenols	GC-MS, FTIR	Nanoemulsion-enhanced formulation	MCF-7, MDA-MB-231	Improved efficacy reported	ROS generation and DNA damage	Salehi et al., (10)
*Plectranthus ecklonii*	Parvifloron D	Abietane diterpenoid	Extraction, HPLC, NMR	First report in TNBC model	MDA-MB-231	0.1–10 µM tested	Anti-migratory and pro-apoptotic	Saraiva et al., (48)
*Spilanthes paniculata*	Spathulenol, Nerolidol	Sesquiterpenes	GC-MS, Molecular docking	First oral cancer stemness study	SCC9	113.95 µg/mL	Apoptosis and inhibition of orosphere formation	Satapathy et al., (30)
*Salvia pisidica*	1,8-Cineole, Camphor, γ-Gurjunene	Monoterpenes/Sesquiterpenes	GC-MS	First anticancer evaluation of endemic Turkish species	MCF-7, Caco-2, HepG2, LnCap	Not reported	Intrinsic and extrinsic apoptosis	Semiz et al., (54)
*Tanacetum sinaicum*	Chrysanthenyl acetate, Thujone, Verbenol	Monoterpenes	GC-MS	First antiviral–anticancer assessment	HeLa, MCF-7	100–400 µg/mL active range	Antiproliferative and anti-metastatic activity	Sheashea et al., (55)
*Ocimum tenuiflorum*	Eugenol, β-Elemene	Phenylpropanoid/Terpene	GC-MS	First EGFR-targeted validation	A549	38.28 µg/mL	Antiproliferative activity	Singh et al., (56)
*Coriandrum sativum*	Decanal, Dodecanal, Phytol	Aldehydes/Terpene alcohol	GC-MS	First EGFR-targeted validation	A549	~45 µg/mL	Antiproliferative activity	Singh et al., (56)
*Origanum onites*	Carvacrol	Monoterpene phenol	GC-MS	First oral administration antitumor study	HT-29, CT26	Not reported	Anti-migratory and pro-apoptotic activity	Spyridopoulou et al., (57)
*Artemisia turanica*	1,8-Cineole, α-Thujone, cis-Chrysanthenol	Oxygenated monoterpenes	Hydrodistillation, GC-MS	First comprehensive anticancer evaluation of Iranian *A. turanica* EO	HeLa	17.67 µg/mL	Selective cytotoxicity; antimutagenic activity	Taherkhani, (58)
*Acokanthera schimperi*	Pregnane steroid (Compound 6)	Steroid	Bioassay-guided isolation, NMR, HRMS	First antiproliferative compound isolated from the species	A427, Dan-G, MCF-7, SiSo	GI50 = 2.3–4.7 µM	G2/M arrest; spindle assembly inhibition	Tesfaye et al., (59)
*Gnidia involucrata*	Novel phorbol ester (Compound 5)	Diterpene ester	Bioassay-guided isolation, NMR, HRMS	Previously unreported phorbol ester	Dan-G pancreatic cancer	GI50 = 0.3 ± 0.05 nM	Early apoptosis induction	Tesfaye et al., (59)
*Seseli bocconei*	Germacrene D	Sesquiterpene	Hydrodistillation, GC-MS	Germacrene D identified as new anticancer candidate	HCT116	Not reported	97% cell death at 250 µg/mL	Vaglica et al., (60)
*Seseli tortuosum* subsp. *maritimum*	Germacrene D-rich EO	Sesquiterpene-rich EO	Hydrodistillation, GC-MS	First report of anticancer activity from endemic Sicilian species	HCT116	Not reported	82% reduction in viability	Vaglica et al., (60)
*Decatropis bicolor*	δ-Cadinene	Sesquiterpene	GC-MS, Molecular docking, Molecular dynamics	First selective MMP-2 inhibitory study	MDA-MB-231	~53.8 µg/mL (reported previously)	Anti-invasive and anti-metastatic activity	Reyes-Vidal et al., (61)
*Cotinus coggygria*	Myricetin, Fisetin, Rutin	Flavonoids	HPLC, LC-MS	First kinetic/mechanistic glioblastoma study	Glioblastoma cell lines	Not reported in abstract	PI3K/Akt and ERK inhibition	Wang et al., (12)
*Toona sinensis*	β-Caryophyllene, Copaene, β-Eudesmene	Sesquiterpenes	Hydrodistillation, GC-MS	First apoptosis-focused evaluation of TSL essential oil	SGC-7901, HepG2, HT29	Not reported	Bax↑, Caspase-3↑, Bcl-2↓	Wu et al., (51)
*Carpesium abrotanoides*	Compound 6 (Sesquiterpene lactone)	Sesquiterpene lactone	Isolation, NMR, MS	First report of protective autophagy induced by sesquiterpene lactones from this species	A549, HepG2, HCT116, MDA-MB-231, CNE2	2.73–7.21 µM	ROS accumulation, G2/M arrest, apoptosis	Yang et al., (52)
*Boswellia serrata*	Monoterpene- and sesquiterpene-rich EO	Terpenoids	Hydrodistillation, GC-MS	First study showing synergy with doxorubicin in TNBC	MDA-MB-231	164.8 µg/mL (24 h); 98.21 µg/mL (48 h)	ROS generation, apoptosis, caspase-3 activation	Zhang et al., (31)
*Podocarpus nagi*	7α,8α-Epoxynagilactone (Compound 1)	Nor-diterpenoid lactone	HRESIMS, IR, NMR, X-ray crystallography	Eight new type-B nagilactones reported	A431	0.208 µM	G1 arrest and apoptosis	Zheng et al., (27)
*Cinnamomum zeylanicum*	(E)-Cinnamaldehyde, Eugenol, E-Caryophyllene	Phenylpropanoids	GC-MS	First comprehensive evaluation of commercial bark EO against colorectal cancer	HCT116	Not reported	Apoptosis, G1 arrest, Cyclin D↓, p-AKT↓	Živković et al., (53)

### 3.3 Anticancer activities of bioactive compounds from underexplored aromatic plants

Collectively, preclinical evidence suggests that underexplored aromatic plants are promising sources of anticancer bioactive compounds. However, these findings from cellular and animal studies require validation in well-designed human clinical trials before clinical application can be considered. The identified phytochemicals displayed diverse biological activities, including inhibition of cell proliferation, induction of apoptosis, suppression of metastasis, regulation of oxidative stress, and modulation of key signaling pathways involved in tumour progression. As depicted in **Table 2** below, the anticancer potential of these agents was determined using various cancer models such as breast, colorectal, lung, stomach, liver, prostate, ovarian, mouth, cervix, pancreatic, and brain cancers.

#### 3.3.1 Activity against breast cancer models

Breast cancer was one of the most commonly studied cancer types. Several aromatic plant-derived compounds displayed outstanding cytotoxic and antiproliferative effects on both hormone-dependent and triple-negative breast cancer cell lines. *Pallenis spinosa* essential oil shows antitumour activity in human breast cancer MCF-7 cells *via* the alteration of Bax, Bcl-2, Cyclin D1, and CDK4 expression (28). Similarly, thymol and carvacrol-rich preparations from *Thymus vulgaris* significantly inhibited breast tumour growth in both *in vitro* and *in vivo* models, highlighting their therapeutic potential (13). Glucobrassicin-loaded liposomal formulations showed significant anticancer activity against breast cancer cells by inducing ROS-mediated apoptosis and inhibiting TOP2A, suggesting that formulation-based strategies can enhance the therapeutic efficacy of plant-derived compounds (63). Also, parvifloron D, isolated from *Plectranthus ecklonii*, had significant effects on the migration and apoptosis of triple-negative breast cancer cells (48). Similarly, the extract of *Boswellia serrata* was effective against triple-negative breast cancer cells as well. Specifically, there is an interaction between this essential oil and doxorubicin, suggesting that it may be appropriate as an adjuvant therapy to reduce chemotherapy-induced toxicity (31).

#### 3.3.2 Activity against colorectal cancer models

Another top area of study was colorectal carcinoma (colon cancer), where so many compounds showed strong growth-inhibitory potential towards HCT116, HT-29, and CT26 colorectal cancer models. Carvacrol-rich *Origanum onites* essential oil exhibited significant antiproliferative action and effectively suppressed tumour growth in a CT26 murine model, providing important *in vitro* evidence supporting its therapeutic potential (57). Likewise, phytochemicals from *Teucrium ramosissimum* enhanced TRAIL-mediated apoptosis and sensitized colorectal cancer cells to programmed cell death through modulation of DR4, DR5, CHOP, and MAPK signaling pathways (69).

The essential oil of *Cedrus atlantica* demonstrated significant cytotoxicity against colorectal cancer cells through activation of caspase-dependent apoptotic pathways and inhibition of PI3K signaling (68). In addition, sesquiterpenoid-rich oils from the endemic *Seseli genus* demonstrated highly significant activity on the HCT116 cancer cell line, and this finding indicates that valuable aromatic members of the Apiaceae may be used as potential sources for anticancer drugs (60).

#### 3.3.3 Activity against lung cancer models

Some studies indicated promising activity in combating non-small cell lung cancer (NSCLC). The essential oil from *Plectranthus amboinicus* was found to be highly cytotoxic to H1299 lung cancer cells while being relatively non-toxic to normal cells (33). Similarly, aspiletrein A, isolated from *Aspidistra letreae* emerged as a particularly promising compound for NSCLC treatment. The compound markedly decreased the migration and invasion in lung cancer cells by the repression of Akt signaling, thus could be promising for blocking the development of metastasis (25). Essential oil constituents from *Chrysopogon zizanioides* demonstrated antiproliferative activity against lung cancer cells and were predicted to target AKT1 and STAT3 signaling pathways through network pharmacology analyses (20). These findings suggest that compounds derived from aromatic plants may offer promising therapeutic options for the treatment of aggressive lung cancers.

#### 3.3.4 Activity against liver and gastric cancer models

Other compounds show effective inhibition in models for HCC and GC. For instance, PTCP-1, a new pentacyclic triterpenoid isolated from *Leptopus lolonum* displays remarkable toxicity to HepG2 cells and leads to apoptosis *via* perturbation of pathways of MAPK and Akt (24). Likewise, 6,7-dehydroroyleanone displayed strong antiproliferative effects against liver cancer cells through activation of intrinsic apoptotic pathways (38). Essential oils from *Meriandra dianthera* and *Plectranthus cylindraceus* also demonstrated notable activity against HepG2 cells, further supporting the therapeutic relevance of aromatic plant-derived terpenoids (46, 47).

In gastric cancer models, bornyl acetate isolated from *Amomum villosum* suppressed proliferation and migration through targeting HMOX1-associated pathways (40). Likewise, the essential oil of *Pistacia atlantica*, which is rich in *α*-pinene, has shown growth inhibitory effects against gastric cancer cells through apoptosis and oxidative stress induced processes (49).

#### 3.3.5 Activity against prostate, oral, ovarian, and other cancer types

In addition to those listed above, some major cancers were also shown to be susceptible to compounds from aromatic plants. Furthermore, *Hedychium spicatum* oil was shown to have a selectivity index favouring prostatic carcinoma against normal fibroblasts, indicating favourable therapeutic selectivity (21). Essential oils from *Myrciaria tenella* and *Spilanthes paniculata* demonstrated strong antiproliferative activity in oral cancer models and inhibited the formation of cancer stem cell-like spheroids, indicating their potential to reduce tumour recurrence (30, 65).

Several studies also reported activity against ovarian cancer cells. Essential oil from *Heracleum persicum* significantly reduced the *via*bility of OVCAR-3 ovarian cancer cells, demonstrating concentration-dependent cytotoxicity (66). In addition, volatile compounds derived from *Lantana camara* exhibited selective anticancer activity against breast and ovarian cancer models (70).

Remarkable activity was also reported against glioblastoma and neuroblastoma cells. Flavonoids isolated from *Cotinus coggygria* demonstrated substantial antiglioblastoma activity both *in vitro* and *in vivo* through inhibition of PI3K/Akt and ERK signaling pathways (12). Similarly, *Pinus sylvestris* essential oil induced apoptosis in glioblastoma cells through ROS-mediated mitochondrial dysfunction (41).

#### 3.3.6 Comparative potency of identified compounds

Comparison of IC_50_ and GI_50_ values revealed substantial variation in anticancer potency among the identified compounds. The most potent activities were observed for the phorbol ester isolated from *Gnidia involucrata* (GI_50_: 0.3 nM), 7*α*,8*α*-epoxynagilactone from *Podocarpus nagi* (IC_50_: 0.208 μM), and several sesquiterpene lactones isolated from *Carpesium abrotanoides* (27, 52, 59).

**Figure 3:**
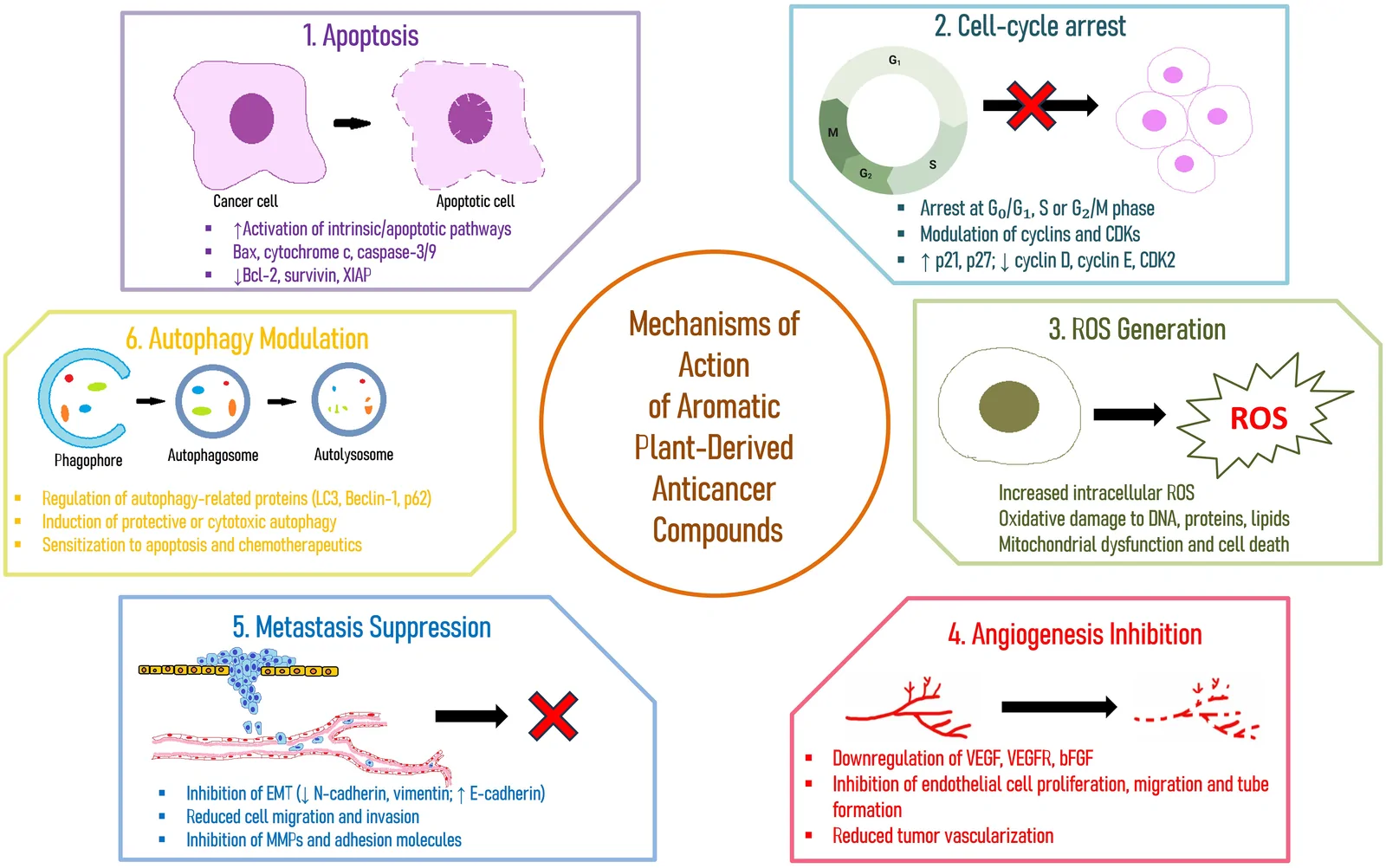
Mechanisms of action of aromatic plant-derived anticancer compounds.

Overall, the evidence summarized in **Table 2** demonstrates that underexplored aromatic plants produce structurally diverse phytochemicals with significant anticancer activities across multiple cancer types. The wide range of effects exerted by these herbs, as well as their promising activity and the limited translational data available, suggest that these herbs are a potential source of novel anticancer drug leads (**Figure 3**). While the preceding section describes the observed anticancer effects, understanding the molecular mechanisms underlying these activities is essential for evaluating their therapeutic relevance.

### 3.4 Molecular mechanisms underlying anticancer activity

According to the literature reviewed here, bioactive molecules derived from underexplored aromatic plants have demonstrated anticancer activities in preclinical models through various mechanisms targeting cancer hallmarks. Rather than acting through a single pathway, many phytochemicals demonstrated multitarget activities involving apoptosis induction, cell-cycle arrest, oxidative stress modulation, inhibition of angiogenesis, suppression of metastasis, regulation of autophagy, and modulation of immune responses (**Table 3**). These pleiotropic actions are especially beneficial in cancer therapy because tumour progression involves multiple, interconnected signaling pathways.

#### 3.4.1 Apoptosis induction

Apoptosis induction was the most frequently reported mechanism among the included studies. Numerous compounds triggered programmed cell death through activation of intrinsic mitochondrial pathways and caspase-dependent signaling cascades.

Essential oil from *Hedychium spicatum* induced apoptosis in prostate cancer cells through increased expression of Bax, Bak, caspase-3, caspase-8, and caspase-9, accompanied by downregulation of anti-apoptotic proteins Bcl-2 and Bcl-xL (21). Similarly, *α*-pinene-rich *Pinus sylvestris* essential oil promoted mitochondrial dysfunction and apoptosis in glioblastoma and neuroblastoma cells through modulation of Bax/Bcl-2 signaling and activation of caspase-3 and caspase-9 (41). Several isolated compounds also exhibited strong pro-apoptotic activities. PCPT-1 from *Leptopus lolonum* induced apoptosis in HepG2 cells by activating p53-mediated pathways and suppressing MAPK/Akt signaling (24). Likewise, paeoniflorigenone isolated from *Paeonia suffruticosa* promoted apoptosis while simultaneously regulating autophagy-related pathways in head and neck squamous cell carcinoma cells (26). Likewise, epunctanone from *Garcinia epunctata* demonstrated potent cytotoxic activity against multidrug-resistant cancer cells by inducing apoptosis and ferroptosis, suggesting a potential role in overcoming therapeutic resistance (23). The same types of apoptosis were also induced by treatment with essential oils of *Pallenis spinosa*, *Toona sinensis, Pistacia atlantica, and Meriandra dianthera* (28, 47, 49, 51).

#### 3.4.2 Cell cycle arrest and growth inhibition

Disruption of the cell cycle is a defining feature of cancer, and several aromatic plant-derived compounds suppressed tumour growth by inducing cell-cycle arrest.

The essential oil of *Pallenis spinosa* induced a G0/G1 arrest by modulating Cyclin D1, CDK4, and p21 expression, resulting in suppression of breast cancer cell proliferation (28). Similarly, nagilactone derivatives isolated from *Podocarpus nagi* induced G1-phase arrest in epidermoid carcinoma cells, contributing to their potent antiproliferative activity (27). Compound 6 isolated from *Carpesium abrotanoides* caused G2/M arrest accompanied by ROS accumulation and autophagy induction in multiple cancer cell lines (52). Likewise, newly identified pregnane steroid derivatives from *Acokanthera schimperi* disrupted mitotic spindle formation, resulting in growth inhibition and arrest at the G2/M checkpoint (59). This work shows that plant-derived aromatic phytochemicals can disrupt cancer cell cycle progression through various checkpoints, thus hindering unrestrained proliferation.

#### 3.4.3 Oxidative stress and ROS-mediated cytotoxicity

ROS have a two-faced role in carcinogenesis. Although moderate amounts of ROS promote cancer development, the overproduction of ROS causes cellular damage and apoptosis. The majority of agents described below were able to take advantage of this weakness through ROS-induced cytotoxicity.

Diosmetin demonstrated significant anticancer activity through ROS generation, leading to oxidative stress-induced apoptosis and tumour suppression in experimental cancer models (44). Similarly, compound 5o derived from *Polyalthia longifolia* promoted ROS accumulation, DNA damage, and activation of caspase-dependent apoptotic pathways in multidrug-resistant colon cancer cells (45). Essential oils from *Boswellia serrata*, *Hedychium spicatum*, and *Pinus sylvestris* also induced oxidative stress-mediated apoptosis through mitochondrial dysfunction and disruption of redox homeostasis (21, 31, 41). Selective susceptibility of cancer cells to the generation-promoting ROS might represent one mechanism behind the anticancer activities of the phytochemicals.

#### 3.4.4 Inhibition of angiogenesis

Angiogenesis plays a pivotal role in tumour growth and metastasis. Several studies have shown that plant aromatics possess anti-angiogenic activity. Nanoencapsulated essential oil from *Trachyspermum ammi* significantly inhibited angiogenesis in the chick chorioallantoic membrane model while simultaneously suppressing colon cancer cell proliferation (35). Likewise, *Thymus vulgaris* and *Cinnamomum zeylanicum* extracts reduced expression of vascular endothelial growth factor (VEGF), thereby limiting neovascularization and tumour progression in breast cancer models (13, 14). These results suggest that interference in tumour vascularization is another pathway underlying the anti-cancer effects of aromatic phytochemicals.

#### 3.4.5 Suppression of migration, invasion, and metastasis

Among several compounds reported in this review article with potent anti-metastatic properties, the most significant issue and causes of cancer death today is its ability of metastatic expansion.

Aspiletrein A, isolated from *Aspidistra letreae*, significantly reduced migration and invasion of non-small-cell lung cancer cells through inhibition of Akt signaling (25). Likewise, δ-cadinene (isolated from *Decatropis bicolor*) inhibited metastasis progression by regulating MMP-2-linked pathways and suppressing cellular invasion (61). Parvifloron D, isolated from *Plectranthus ecklonii*, has also been shown to inhibit metastasis in triple-negative breast cancer cells (48). Together, these results demonstrate the potential anti-cancer, anti-disseminative, and anti-metastatic capacity of phytochemicals isolated from aromatic plants.

#### 3.4.6 Regulation of autophagy and alternative cell death pathways

Several bioactive compounds were found to modulate multiple programmed cell death pathways, including apoptosis, autophagy, ferroptosis, and necroptosis. Paeoniflorigenone stimulated both apoptosis and autophagy while inhibiting necroptotic signaling in head and neck cancer cells, suggesting multiple mechanisms involved in controlling cell fate determination (26). Compound 6 isolated from *Carpesium abrotanoides* promoted autophagy by inducing reactive oxygen species (ROS) accumulation and G2/M cell cycle arrest (52). Similarly, epunctanone effectively induced both ferroptosis and apoptosis in multidrug-resistant cancer cells (23). The capacity for activation of multiple types of programmed cell death by these compounds may provide certain advantages in circumventing resistance to current apoptosis targeted therapies.

#### 3.4.7 Immunomodulatory effects

Developing evidence indicates that some plant aromatic-derived compounds might alter antitumour immune reactions. Carvacrol-rich *Origanum vulgare* subsp. *Hirtum* essential oil improved immune-mediated tumor suppression *via* higher expression of interferon-gamma (IFN-gamma), tumor necrosis factor-alpha (TNF-alpha), granzyme B, and caspase-3 in test tumor models (34). Likewise, the anti-inflammatory effects of several essential oils were associated with the suppression of pro-inflammatory mediators, which may contribute to their anticancer activities by modulating the tumour microenvironment. Although immunomodulatory mechanisms remain relatively underexplored, these findings suggest an additional therapeutic dimension for aromatic plant-derived compounds.

**Figure 4:**
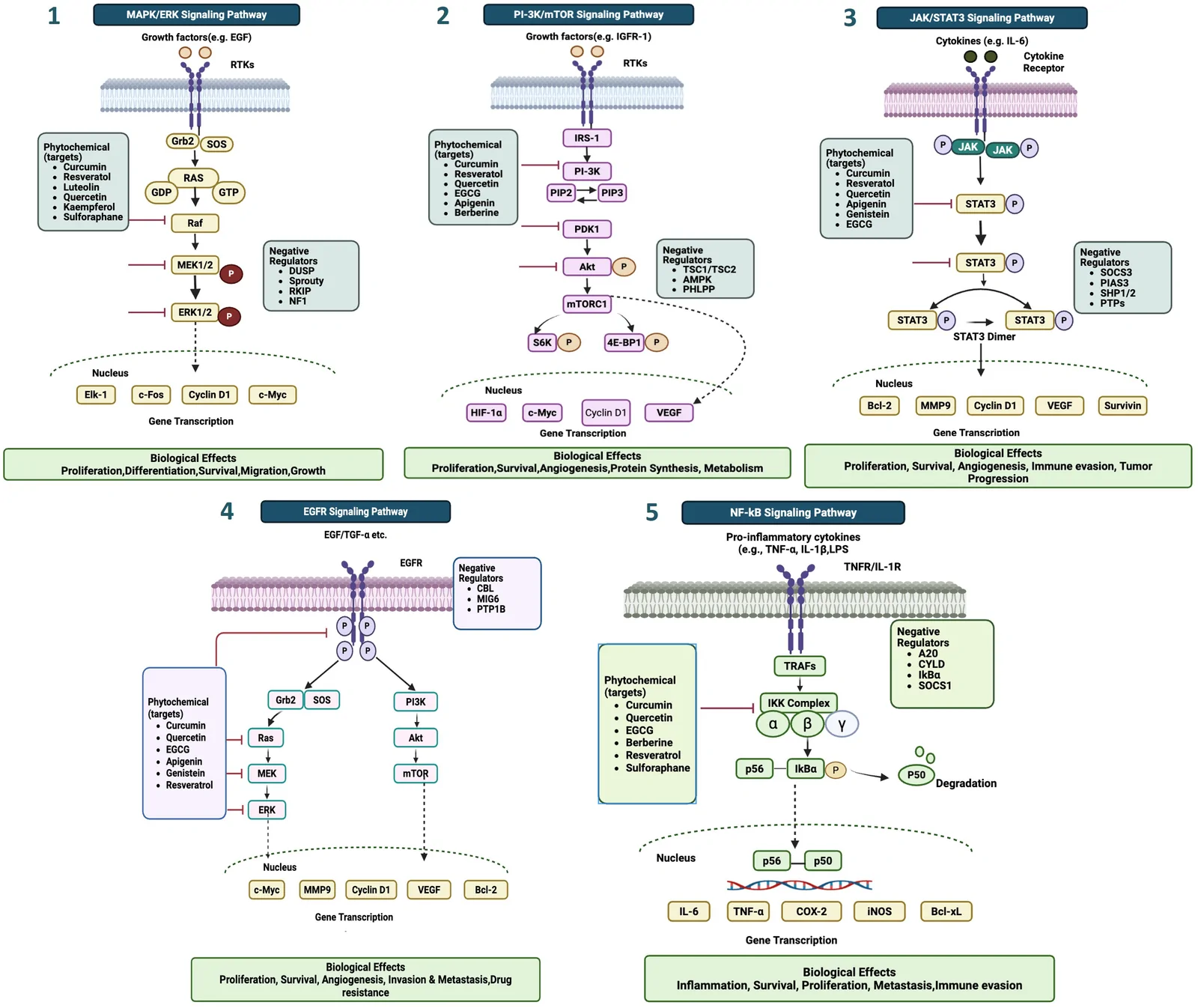
Molecular signaling pathways targeted by phytochemicals.

Overall, the mechanistic evidence summarized in **Table 3** demonstrates that bioactive compounds from underexplored aromatic plants exert anticancer effects through multiple complementary pathways. Their ability to modulate apoptosis, cell-cycle progression, oxidative stress, angiogenesis, metastasis, autophagy, and immune regulation highlights their potential as multitarget anticancer candidates in preclinical studies. However, whether these mechanisms translate into clinical benefit remains to be established in human studies (**Figure 4**). Many of the biological effects described above are mediated through the modulation of specific intracellular signaling pathways, which are discussed in the following section.

**Table 3:** Mechanistic Insights, Translational Evidence, and Clinical Potential of Identified Compounds

Compound	Mechanism of Action	Molecular Targets	Biological Effect	In vivo Validation	Pharmacokinetic/Toxicity Data	Clinical Potential	Reference
α-Pinene, 1,8-Cineole, Limonene (*Myrtus communis* EO)	Induction of apoptosis and oxidative stress	Not specifically identified	Cytotoxicity against P815 mastocytoma cells	No	Not reported	Moderate; requires mechanistic validation	Harassi et al. (62)
Glucobrassicin (*Tinospora cordifolia*)	ROS-mediated apoptosis and migration inhibition	TOP2A (Topoisomerase IIα), Bcl-2, Caspase-3, Cytochrome-c	Antiproliferative and pro-apoptotic effects	No	Nano-liposomal formulation (~153 nm) improved delivery	High; promising breast cancer candidate	Muhasina et al. (63)(2025)
Camphor, Borneol, Artemisia ketone derivatives (*Artemisia serotina*)	Cytotoxicity induction	Not reported	Anticancer and antimicrobial activities	No	Not reported	Moderate	Kadyrbay et al. (64)
Thymol-rich EO (*Plectranthus amboinicus*)	Cell death induction	Not investigated	Strong antiproliferative activity in H1299 cells	No	Low toxicity in Vero cells	High potential for lung cancer studies	Abdalla et al. (33)
Carvacrol-rich EO (*Origanum vulgare* ssp. *hirtum*)	Immune-mediated apoptosis	Caspase-3, IFN-γ, TNF-α, Granzyme-B	Tumor suppression and immune activation	Yes (CT26/BALB-c model)	No toxicity reported	High translational potential	Aindelis et al. (34)
Thymol-rich nanoencapsulated EO (*Trachyspermum ammi*)	Apoptosis and antiangiogenesis	Bax, Caspase pathways	Colon cancer inhibition	CAM angiogenesis model	Nanoformulation improved stability	High	Almnhawy et al. 35)
Nerolidol (*Jasonia glutinosa*)	Apoptosis and anti-inflammatory activity	NO, PGE2 signaling	Selective cytotoxicity against MCF-7 and HepG2	No	Genotoxicity assay showed safety up to 100 µg/mL	High	Baraich et al. (36)
Menthone derivatives (*Mentha aquatica*)	G2/M arrest and apoptosis	COX-2, HRAS-associated pathways	Chemoprevention of skin carcinogenesis	Yes (DMBA/TPA mouse model)	No toxicity reported	High chemopreventive value	Chang et al. (37)
6,7-Dehydroroyleanone	Intrinsic apoptosis induction	Caspase-dependent pathways	Potent HepG2 cytotoxicity	No	Not reported	High; lead diterpenoid candidate	Chen et al. (38)
β-Caryophyllene, Germacrene D	Tumor growth suppression	Not specifically identified	In vitro and in vivo antitumor effects	Yes (Sarcoma-180 model)	No significant toxicity reported	High	Lima et al. (39)
Bornyl acetate (*Amomum villosum*)	Suppression of proliferation and migration	HMOX1	Anti-gastric cancer activity	No	Molecular docking supports binding	High	Deng et al. (40)
α-Pinene, β-Pinene-rich EO (*Pinus sylvestris*)	ROS-mediated mitochondrial apoptosis	Bax, Bcl-2, Caspase-3, Caspase-9	Cytotoxicity against glioblastoma and neuroblastoma cells	No	Not reported	High	Dervişoğlu (41)
Eucalyptol, α-Pinene, Camphor (*Rosmarinus officinalis*)	Cytotoxicity induction	Not reported	Colorectal cancer inhibition	No	Low toxicity toward HaCaT cells	Moderate–High	Dolghi et al. (42)
Caryophyllene oxide, α-Pinene (*Myrciaria tenella*)	Predicted enzyme inhibition (docking)	Protein targets identified by docking	Oral cancer cytotoxicity	No	Not reported	Moderate	Ferreira et al. (65)
Hexyl butyrate, Octyl butyrate derivatives (*Heracleum persicum*)	Concentration-dependent cytotoxicity	Not investigated	OVCAR-3 ovarian cancer inhibition	No	Not reported	High	Ghavam (66)
β-Terpineol, δ-Cadinene (*Decatropis bicolor*)	Intrinsic apoptosis pathway	Bax, Caspase-9, Caspase-3	Breast cancer cell apoptosis	No	Selective toward cancer cells	High	Estanislao Gómez et al. (67)
β-Himachalene, α-Himachalene (*Cedrus atlantica*)	Mitochondrial apoptosis	PI3Kγ, Caspase-3/7	Cytotoxicity against melanoma and colorectal cancer	No	Low toxicity on HaCaT cells	High	Gruin et al. (68)
*Teucrium ramosissimum* phytocomplex	TRAIL sensitization and cell cycle arrest	DR4, DR5, JAK/STAT, MAPK, CHOP, SP1	Enhanced apoptosis in HCT116 cells	Yes (LPS-induced colon carcinogenesis)	Not reported	Very High	Guesmi et al. (69)
Volatile compounds from *Lantana camara* flowers	Antiproliferative activity	Not fully characterized	Selective activity against breast cancer cells	No	Not reported	Moderate	El Hajj et al. (70)
Glucobrassicin	TOP2A inhibition and ROS-mediated apoptosis	TOP2A	Anti-breast cancer activity	No	Nanoformulation enhances bioavailability	High	Muhasina et al. (63)(2025)
Diosmetin	ROS-mediated apoptosis and cell cycle arrest	ROS pathway, Akt signaling	Antiproliferative activity; tumor growth suppression	Yes (EAC mouse model)	High GI absorption; non-toxic to erythrocytes	High	Raza et al. (44)
Khusimol, β-Eudesmol, α-Vetivone, Rosifoliol (*Chrysopogon zizanioides* EO)	Growth inhibition and pathway modulation	AKT1, STAT3	Antiproliferative activity in CRC and lung cancer	No	Favorable ADMET; low predicted toxicity	High	Karousa et al. (20)
Thymol, Carvacrol-rich *Thymus vulgaris* extract	Apoptosis, antiangiogenesis, epigenetic regulation	Bax, Caspase-3, VEGF, Ki67	Mammary tumor suppression	Yes (4T1 and rat mammary carcinoma models)	No significant toxicity reported	High	Kubatka et al. (13)
Cinnamaldehyde, Eugenol (*Cinnamomum zeylanicum*)	Apoptosis induction and proliferation inhibition	Bax, Caspase-3, VEGF, Ki67	Breast cancer suppression	Yes (rat and mouse models)	No major toxicity observed	High	Kubatka et al. (14)
Compound 5o (maleimide diterpene derivative)	ROS generation, DNA damage, caspase-dependent apoptosis	Caspases, ROS signaling, cell-cycle proteins	Growth inhibition of multidrug-resistant colon cancer	Yes (Balb/c xenograft model)	Oral bioavailability; t½ ≈ 3 h; >80% stability; no observable toxicity	Very High	Kumar et al. (45)
Geranial, Neral, α-Curcumene (*Aloysia citriodora* EO)	Cytotoxicity induction	Not fully elucidated	Potent anti-melanoma activity	No	Not reported	Moderate–High	Al-Maharik et al. (29)
Epunctanone	Apoptosis and ferroptosis induction	Caspases, ROS, mitochondrial membrane potential	Cytotoxicity against MDR cancer cells	No	Not reported	High	Mbaveng et al. (23)
Maaliol-rich *Plectranthus cylindraceus* EO	Cytotoxicity induction	Not identified	Strong activity against HeLa, HepG2 and HT-29	No	Not reported	Moderate	Mothana et al. (46)
Camphor, 1,8-Cineole (*Meriandra dianthera* EO)	Apoptosis induction	Bcl-2 family proteins	Cytotoxicity against MCF-7 and HepG2	No	Not reported	Moderate	Mothana et al. (47)
Aspiletrein A	Anti-migration and anti-invasion	pAkt/Akt signaling pathway	Antimetastatic activity in NSCLC	No	Not reported	High	Nguyen et al. (25)
β-Caryophyllene, α-Humulene-rich EO (*Aegle marmelos*)	Antiproliferative activity	Not fully characterized	Potent inhibition of pancreatic, ovarian, colon and lung cancer cells	Biodistribution study in mice	Distribution quantified in organs after oral administration	High	Pant et al. (71)
Paeoniflorigenone	Apoptosis, autophagy induction, necroptosis suppression	PI3K/Akt/mTOR/p70S6K, RIP, MLKL, LC3	Anti-HNSCC activity and anti-metastatic effects	No	Compound purity 98.9%; toxicity not reported	High	Park et al. (26)
PCPT-1	G1 arrest and apoptosis	p53, MAPK, Akt pathways	Cytotoxicity against HepG2	No	Low toxicity suggested for PCPTs	High	Qi et al. (24)
Thymoquinone	Cytotoxicity and apoptosis induction	Multiple cancer-related targets (in silico validated)	Antileukemic activity	No	Lower toxicity than conventional chemotherapy	High	Ravi et al. (43)
*Hedychium spicatum* EO	ROS-mediated mitochondrial apoptosis	Caspase-3, Caspase-8, Caspase-9, Bax, Bak, Bcl-2, Bcl-xL	Selective activity against prostate cancer cells	No	Selective toward cancer cells over normal fibroblasts	High	Ray et al. (21)
Parvifloron D	Apoptosis and inhibition of migration/invasion	Apoptotic signaling pathways	Anti-TNBC activity	No	Active at low micromolar concentrations	High	Saraiva et al. (48)
α-Pinene-rich *Pistacia atlantica* EO	Apoptosis induction	Bax, Bcl-2	Growth inhibition of gastric cancer cells	No	Not reported	Moderate–High	Saeedi et al. (49)
Thymol, Carvacrol, Citral, Eugenol, Geraniol	Caspase-dependent apoptosis	Bax, Bak, Caspase-7, Caspase-9, Bcl-2, pERK, pAkt	Strong antiproliferative activity in MCF-7 and T24	No	In silico interaction and favorable profiles	High	Saini et al. (50)
*Pallenis spinosa* Flower EO	Caspase-dependent and independent apoptosis; G0/G1 arrest	Cyclin D1, CDK4, p21, Bax, Bcl-2	Potent breast cancer cytotoxicity	No	Selective toward cancer cells over normal cells	Very High	Saleh et al. (28)
Zataria EO Nanoemulsion	ROS generation, mitochondrial dysfunction, DNA damage, apoptosis	Mitochondria, genomic DNA	Enhanced cytotoxicity in resistant breast cancer spheroids	Spheroid model	Nanoemulsion improved stability and efficacy	Very High	Salehi et al. (10)
*Boswellia serrata* Essential Oil	ROS-mediated apoptosis, caspase-3 activation, nuclear damage	ROS, Caspase-3	Antiproliferative and apoptosis-inducing effects against MDA-MB-231 breast cancer cells	No	Synergistic with doxorubicin; reduced effective chemotherapy dose	High	Zhang et al. (31)
*Spilanthes paniculata* Flower Essential Oil	Induction of apoptosis and inhibition of cancer stem-cell-like spheroid formation	p53 (in silico), apoptosis pathways	Cytotoxicity against SCC9 oral cancer cells	No	Spathulenol and nerolidol showed favorable ADMET and GI absorption	High	Satapathy et al. (30)
*Tanacetum sinaicum* Essential Oil	Autophagy induction, DNA damage, antiproliferative and anti-metastatic activity	HPV-16/18 suppression, autophagic pathways	Inhibition of HeLa and MCF-7 cell proliferation and metastasis	No	Dose-dependent cytotoxicity; antiviral activity	High	Sheashea et al. (55)
*Artemisia turanica* Essential Oil	Selective cytotoxicity toward cancer cells	Not specifically identified	Cytotoxicity against HeLa cells with minimal effect on lymphocytes	No	IC50 HeLa: 17.67 μg/mL vs. normal lymphocytes: 3291 μg/mL	Moderate–High	Taherkhani (58)
Phorbol ester (Compound 5) and Pregnane derivative (Compound 6)	Early apoptosis; G2/M arrest via spindle disruption	Mitotic spindle apparatus	Potent antiproliferative activity across multiple cancer cell lines	No	GI50 as low as 0.3 nM (compound 5)	Very High	Tesfaye et al. (59)
Germacrene D from *Seseli* spp. Essential Oils	Cytotoxicity induction	Not elucidated	Significant inhibition of HCT116 colon cancer cells	No	Not reported	Moderate–High	Vaglica et al. (60)
δ-Cadinene (*Decatropis bicolor*)	Anti-invasive and selective cytotoxic activity	MMP-2, adhesion/invasion proteins	Reduced viability and invasion of MDA-MB-231 cells	No	Minimal effect on normal MCF10A cells	High	Reyes-Vidal et al. (61)
Myricetin-rich flavonoids from *Cotinus coggygria*	Mitochondrial apoptosis; PI3K/Akt and ERK inhibition	PI3K/Akt, ERK, caspases	Antiglioblastoma activity	Yes (orthotopic mouse model)	Demonstrated in vivo efficacy	Very High	Wang et al. (12)
*Toona sinensis* Leaf Essential Oil	Bax upregulation, Caspase-3 activation, Bcl-2 suppression	Bax, Bcl-2, Caspase-3	Apoptosis induction in SGC-7901 gastric cancer cells	No	High sesquiterpene content linked to activity	High	Wu et al. (51)
*Ocimum tenuiflorum* Essential Oil	EGFR inhibition (in silico), antiproliferative activity	EGFR	Cytotoxicity against A549 lung cancer cells	No	IC50 = 38.28 μg/mL; favorable docking scores	Moderate–High	Singh et al. (56)
Carvacrol-rich *Origanum onites* Essential Oil	Apoptosis induction and migration inhibition	Apoptosis-related pathways	Colon cancer growth suppression	Yes (CT26 mouse model)	Effective after oral administration	Very High	Spyridopoulou et al. (57)
Type B Nagilactone (Compound 1)	Apoptosis induction and G1 cell-cycle arrest	Cell-cycle regulators	Potent cytotoxicity against A431 carcinoma cells	No	IC50 = 0.208 μM	High	Zheng et al. (27)
Cinnamon Bark Essential Oil (*Cinnamomum zeylanicum*)	Apoptosis induction, G1 arrest, AKT suppression	Cyclin D, p-AKT	Selective antitumor activity in HCT116 colorectal cancer cells	No	Sparing of normal MRC-5 cells	High	Živković et al. (53)
*Salvia pisidica* Essential Oil	Intrinsic and extrinsic apoptosis pathways	Apoptosis-related genes	Cytotoxicity against breast, colon, liver and prostate cancer cells	No	Not reported	High	Semiz et al. (54)
Sesquiterpene Lactone (Compound 6) from *Carpesium abrotanoides*	ROS accumulation and G2/M arrest leading to autophagy	ROS signaling, cell-cycle machinery	Broad-spectrum cytotoxicity against A549, HepG2, HCT116, MDA-MB-231, CNE2	No	IC50 ≈ 2.73–7.21 μM	High	Yang et al. (52)

### 3.5 Modulation of cancer-related signaling pathways

A prominent finding from the included studies is that aromatic plant-derived phytochemicals can regulate multiple cancer-associated signaling pathways involved in cell proliferation, survival, angiogenesis, metastasis, and therapeutic resistance. Many phytochemicals have multitarget activities. Unlike common anticancer drugs, which target just one signaling pathway, phytochemicals can regulate multiple interconnected pathways simultaneously. For example, PI3K/Akt/mTOR, MAPK, STAT3, EGFR and NF-*k*B signaling pathways were the most widely observed targeted molecular pathways based on our findings (**Table 3**).

#### 3.5.1 PI3K/Akt/mTOR signaling pathway

The PI3K/Akt/mTOR signaling pathway plays a central role in regulating tumour growth, metabolism, cell survival, and therapeutic resistance. Dysregulation of this pathway is commonly observed in breast, lung, colorectal, prostate, and head and neck cancers. Several phytochemicals identified in this review demonstrated the ability to suppress PI3K/Akt/mTOR signaling, thereby inhibiting cancer cell proliferation and promoting apoptosis. Paeoniflorigenone isolated from *Paeonia suffruticosa* significantly inhibited PI3K/Akt/mTOR signaling while simultaneously inducing apoptosis and autophagy in head and neck squamous cell carcinoma cells (26). Similarly, flavonoids isolated from *Cotinus coggygria* suppressed PI3K/Akt signaling in glioblastoma models, contributing to reduced tumour growth and enhanced apoptosis (12). Such results emphasize the significance of PI3K/Akt/mTOR pathway suppression as a major mechanism responsible for anticancer properties of aromatic plant components.

#### 3.5.2 MAPK signaling pathway

Mitogen-activated protein kinase (MAPK) pathway controls cellular growth, differentiation, stress, and apoptosis. Deregulation of MAPK signaling can mediate cancer progression and therapy resistance. Several compounds reviewed here demonstrated significant modulation of MAPK-associated pathways.

PCPT-1, a novel pentacyclic triterpenoid isolated from *Leptopus lolonum*, induced apoptosis through inhibition of MAPK and Akt signaling pathways in hepatocellular carcinoma cells (24). Likewise, phytochemicals from *Teucrium ramosissimum* enhanced TRAIL-mediated apoptosis through regulation of MAPK signaling components, thereby increasing sensitivity of colorectal cancer cells to programmed cell death (69). The ability of these compounds to modulate MAPK signaling suggests their potential utility in targeting cancers characterized by dysregulated proliferative signaling.

#### 3.5.3 STAT3 signaling pathway

Signal transducer and activator of transcription 3 (STAT3) is a key oncogenic transcription factor that promotes tumour growth, angiogenesis, immune evasion, and metastatic progression. Persistent STAT3 activation is associated with poor prognosis in several malignancies.

Network pharmacology and molecular docking analyses identified AKT1 and STAT3 as major molecular targets of the essential oil of *Chrysopogon zizanioides* (vetiver) (20). The major constituents, including khusimol, *α*-vetivone, and rosifoliol, demonstrated strong binding affinity toward STAT3-associated targets, suggesting a mechanistic basis for their observed antiproliferative effects. While currently available data is limited in terms of the extent of its confirmation by *in vitro* studies, these initial results provide a baseline idea of the impact of STAT3 inhibition on the antitumour capabilities of plants that contain aromatic compounds.

#### 3.5.4 EGFR and receptor-mediated signaling

In lung, colorectal and head and neck cancers, overexpression of the EGFR pathway is highly frequent, where it serves as an important therapeutic target. Several aromatic plant-derived compounds have been shown to interact with EGFR-related signaling pathways.

Antiproliferative activity was noted on lung cancers, with oils from *Ocimum tenuiflorum* and *Coriandrum sativum* being particularly effective, along with favourable binding interactions to EGFR (epidermal growth factor receptor) in studies through docking (56). These findings suggest that aromatic plant constituents may function as natural modulators of receptor-mediated signaling pathways involved in tumour progression. Further mechanistic and *in vitro* research is needed to further validate these computational predictions.

#### 3.5.5 NF-κB and inflammatory signaling

The nuclear factor-kappa B (NF-κB) signaling pathway is a central mediator of chronic inflammation, cancer progression, metastasis, and resistance to apoptosis. Although relatively few studies directly examined NF-κB inhibition, several phytochemicals displayed anti-inflammatory and immunomodulatory properties that are likely associated with the suppression of NF-κB-mediated signaling. Carvacrol-rich essential oils and several terpenoid compounds demonstrated anti-inflammatory effects through reduction of pro-inflammatory mediators, suggesting indirect modulation of NF-κB-associated pathways (34). These actions can help suppress tumour-promoting inflammation in the tumour microenvironment.

#### 3.5.6 Emerging molecular targets

Novel therapeutic targets were reported for the compound in this study, other than the classic signaling paths involved in oncogenesis. Bornyl acetate isolated from *Amomum villosum* inhibited gastric cancer progression through targeting heme oxygenase-1 (HMOX1), a protein implicated in oxidative stress regulation and tumour survival (40). Similarly, glucobrassicin-loaded nanoformulations suppressed TOP2A-associated signaling in breast cancer cells, providing a potential strategy for overcoming resistance to conventional therapies (63).

Collectively, these findings demonstrate that phytochemicals from underexplored aromatic plants regulate multiple cancer-related signaling pathways involved in tumour initiation, progression, metastasis, and therapeutic resistance. Their ability to act on multiple targets is an important strength compared with traditional single-target drugs and supports their further preclinical evaluation as potential sources of future anticancer agents. Additional pharmacological investigations and clinical studies are required to confirm their therapeutic value in humans. Although modulation of multiple oncogenic signaling pathways highlights the therapeutic promise of these compounds, successful drug development also depends on their translational feasibility, including efficacy, safety, pharmacokinetics, and formulation strategies.

### 3.6 Translational potential of aromatic plant-derived anticancer compounds

The successful transition of natural products from the laboratory to clinical application is not solely dependent on their biological activity. Factors such as favourable pharmacokinetic properties, demonstrated efficacy, acceptable safety profiles, and comprehensive preclinical and clinical evaluation are all critical for therapeutic development. Although most studies included in this review were limited to *in vitro* investigations, several advanced the translational potential of these compounds through *in vitro* validation, pharmacokinetic assessment, formulation development, and the identification of promising lead molecules. Overall, these findings highlight the potential of phytochemicals from underexplored aromatic plants as preclinical candidates for anticancer drug development. However, the current evidence is derived primarily from *in vitro* and animal studies, while robust clinical evidence in humans remains limited.

#### 3.6.1 *In vitro* validation studies

Research has consistently shown that compounds derived from aromatic plants exhibit anticancer activity in *in vitro* experiments and animal models. For instance, *Origanum onites* essential oil, which is rich in carvacrol, a naturally occurring compound, showed substantial tumour suppression in mice with CT26 colorectal cancer and notably, without causing significant harm to the overall health of animals. Similarly, extracts from common culinary herbs like thyme and cinnamon have demonstrated the ability to slow breast tumour development by working through multiple biological mechanisms triggering cancer cell death, restricting blood vessel formation to tumours, and reducing the cellular damage caused by reactive oxygen species (13, 14).

Additional *in vitro* evidence was reported for flavonoids isolated from *Cotinus coggygria*, which reduced glioblastoma growth in murine models through suppression of PI3K/Akt signaling (12). Furthermore, compound 5o derived from *Polyalthia longifolia* exhibited significant antitumour efficacy against colon cancer xenografts, confirming the therapeutic relevance of its potent *in vitro* activity (45). These findings suggest that certain compounds retain biological activity in animal models and therefore warrant further preclinical investigation, rather than demonstrating clinical efficacy.

#### 3.6.2 Pharmacokinetic and toxicity evidence

One of the major challenges in natural-product drug development is the lack of comprehensive pharmacokinetic and safety data. Most studies reviewed here focused primarily on biological activity, with only a few evaluating absorption, distribution, metabolism, excretion, and toxicity characteristics. Among the identified compounds, compound 5o exhibited favourable pharmacokinetic properties, including improved metabolic stability and oral bioavailability, supporting its potential as a lead candidate for further development (45).

The essential oils of *Hedychium spicatum*, *Plectranthus amboinicus*, and *Aegle marmelos* showed selective cytotoxicity against cancer cells with relatively low toxicity toward normal cell lines (21, 33, 71). Despite this being the case, however, adequate toxicological evaluations have not been done yet. Ongoing and future studies should focus on assessing acute, chronic, and organ-specific toxicities to establish the safety of these compounds.

#### 3.6.3 Nanoformulations and targeted delivery systems

Lack of aqueous solubility, low oral bioavailability, high rate of metabolism and poor accumulation in the tumour mass limit its application. Nanotechnology-based delivery systems may provide new methods to overcome these limitations.

Compared with conventional formulations, nanoencapsulated *Trachyspermum ammi* essential oil exhibited enhanced antiangiogenic and anticancer activities, likely as a result of improved stability and cellular uptake (35). Likewise, nanoemulsions formulated using *Zataria multiflora* essential oil showed enhanced cytotoxicity and bioavailability compared to free essential oil preparations (10). Moreover, glucobrassicin-loaded liposomes also demonstrated enhanced therapeutic efficacy in models of breast cancer owing to improved drug delivery and sustained intracellular activity (63). All these studies together show that nanocarrier systems like liposomes, nanoemulsions, polymeric nanoparticles, and phytosomes can really enhance the effectiveness of compounds derived from aromatic plants and help bring them into clinical use.

#### 3.6.4 Promising lead compounds for drug development

Some phytochemicals discovered through this review are potential candidates for development of anticancer drugs. Among them, epunctanone, isolated from *Garcinia epunctata*, exhibited potent activity against multidrug-resistant cancer cells by inducing both apoptosis and ferroptosis, highlighting its potential to overcome chemotherapy resistance (23). Similarly, PCPT-1 from *Leptopus lolonum* demonstrated significant antiproliferative effects against hepatocellular carcinoma through modulation of the MAPK/Akt signaling pathway (24). Other noteworthy compounds include aspiletrein A from *Aspidistra letreae*, paeoniflorigenone from *Paeonia suffruticosa*, 7*α*,8*α*-epoxynagilactone from *Podocarpus nagi*, and a highly potent phorbol ester isolated from *Gnidia involucrata*, which exhibited subnanomolar antiproliferative activity (25–27, 59). These compounds possess unique structures and demonstrate encouraging preclinical biological activity.

In addition to their inherent anticancer activities, certain phytochemical compounds obtained from aromatic plants have been shown to lower the risk of toxicity associated with chemotherapeutic drugs. In one case, *α*-bisabolol has been observed to mitigate doxorubicin-induced nephrotoxicity through the inhibition of oxidative stress, the NF-κB/MAPK pathway, and apoptotic cell death, while protecting the architecture of the kidney (77). Among the compounds reviewed, compound 5o (maleimide diterpene derivative) has the strongest preclinical evidence, supported by robust mechanistic validation, favourable pharmacokinetics, and *in vivo* antitumour efficacy. In contrast, paeoniflorigenone, PCPT-1, and aspiletrein A show promising mechanistic activity but require further *in vivo*, pharmacokinetic, and toxicological validation. Bornyl acetate and vetiver essential oil are supported mainly by preliminary mechanistic or computational evidence and remain early-stage candidates for further investigation. Although several compounds exhibited comparable cytotoxic potency, translational readiness varied substantially. Potency alone was insufficient to identify the most promising drug candidates because many studies lacked *in vivo* validation, pharmacokinetic characterization, or toxicity assessment. Consequently, compounds supported by multiple complementary lines of evidence were considered to have greater translational potential than compounds evaluated only in cell-based assays. **Table 4** summarizes the comparative preclinical evidence and translational potential of the principal anticancer phytochemicals identified in this review.

**Table 4:** Comparative evaluation of aromatic plant-derived anticancer compounds based on preclinical evidence and translational readiness.

Compound	Plant source	*In vitro* potency	*In vivo* validation	Mechanistic evidence	PK/Toxicity data	Translational readiness	References
Compound 5o	*Polyalthia longifolia*	Very high	Yes	Extensive	Available	High	Kumar et al., (45)
7α,8α-Epoxynagilactone	*Podocarpus nagi*	Very high	Limited	Good	Limited	Moderate	Zheng et al., (27)
PCPT-1	*Leptopus lolonum*	High	Limited	Strong	Limited	Moderate	Qi et al., (24)
Aspiletrein A	*Aspidistra letreae*	High	No	Strong	None	Moderate-Low	Nguyen et al., (25)
Paeoniflorigenone	*Paeonia suffruticosa*	High	Limited	Strong	None	Moderate-Low	Park et al., (26)
Epunctanone	*Garcinia epunctata*	High	Limited	Strong (apoptosis + ferroptosis)	None	Moderate-Low	Mbaveng et al., (23)
Bornyl acetate	*Amomum villosum*	Moderate	No	Preliminary	None	Low	Deng et al., (40)

### 3.7 Challenges, research gaps, and future perspectives

Although the findings of this systematic review highlight the considerable anticancer potential of bioactive compounds from underexplored aromatic plants, several methodological and translational limitations should be considered when interpreting the available evidence. Most included studies reported positive outcomes, raising the possibility of publication bias, as negative or inconclusive findings are less likely to be published. In addition, substantial methodological heterogeneity, including differences in plant authentication, geographical origin, chemotypes, extraction procedures, phytochemical characterization, experimental models, treatment protocols, and outcome measures, limits the reproducibility, comparability, and overall reliability of the reported findings. The current evidence is also dominated by *in vitro* investigations, with relatively few *in vivo* studies and only limited clinical evidence, making it difficult to determine the therapeutic relevance of these promising compounds in human cancer.

A major obstacle to clinical translation is the lack of standardization of plant materials and essential oils. Phytochemical composition is strongly influenced by environmental and biological factors, including geographical origin, soil composition, climatic conditions, developmental stage, harvesting season, and post-harvest processing. Because essential oils comprise complex mixtures of volatile constituents, even minor compositional differences may substantially alter their biological activity, resulting in inconsistent outcomes across studies. Future investigations should therefore emphasize authenticated botanical materials, standardized cultivation and extraction procedures, comprehensive phytochemical profiling, and robust quality-control measures to improve reproducibility and facilitate regulatory acceptance.

Another important challenge is the limited pharmacokinetic and toxicological information available for most aromatic plant-derived phytochemicals. Despite encouraging preclinical efficacy, evidence regarding absorption, distribution, metabolism, excretion (ADME), long-term safety, organ-specific toxicity, and potential drug–drug interactions remain scarce. Furthermore, poor aqueous solubility, limited bioavailability, rapid metabolism, and difficulties in scalable manufacturing continue to hinder clinical development. Addressing these barriers through rigorous pharmacological evaluation, formulation optimization, and standardized preclinical testing will be essential for advancing promising lead compounds toward clinical application.

Significant research opportunities remain in the systematic exploration of aromatic plant biodiversity. Although aromatic plants are widely distributed across biodiversity hotspots such as Northeast India, the Eastern Himalayas, Southeast Asia, Africa, South America, and the Mediterranean region, only a small proportion have been investigated for anticancer activity. Recent discoveries of structurally unique metabolites, including epunctanone, PCPT-1, aspiletrein A, paeoniflorigenone, and novel nagilactones, demonstrate the enormous potential of biodiversity-driven natural product discovery. Future studies should integrate ethnopharmacological knowledge with modern phytochemical, pharmacological, and medicinal chemistry approaches to accelerate the identification of novel lead molecules.

Emerging technologies are expected to play an increasingly important role in the discovery of aromatic plant-based anticancer drugs. Multi-omics approaches, including genomics, transcriptomics, proteomics, and metabolomics, can provide comprehensive insights into biosynthetic pathways, molecular targets, and biomarkers associated with therapeutic response. Likewise, artificial intelligence, machine learning, and network pharmacology offer powerful tools for target prediction, virtual screening, lead optimization, and mechanism-of-action analysis. However, because these approaches were only sparsely represented in the included studies, their application should currently be regarded as a promising research direction rather than established evidence. Computational predictions must be complemented by rigorous experimental validation using appropriate preclinical models before they can contribute meaningfully to clinical drug development.

Advanced drug-delivery technologies also represent an important avenue for future investigation. Many phytochemicals exhibit poor aqueous solubility, limited systemic bioavailability, rapid metabolism, and inadequate tumour accumulation, restricting their therapeutic potential. Nanotechnology-based delivery platforms, including liposomes, polymeric nanoparticles, phytosomes, nanoemulsions, dendrimers, and stimulus-responsive nanocarriers, have shown considerable promise for improving compound stability, controlled release, targeted tumour delivery, and therapeutic efficacy. Future studies should evaluate these delivery systems alongside comprehensive pharmacokinetic, toxicological, and efficacy assessments to maximize the translational potential of aromatic plant-derived bioactive compounds.

Overall, future progress in this field will require coordinated, multidisciplinary efforts integrating biodiversity exploration, phytochemistry, molecular pharmacology, computational biology, and translational oncology. Standardized experimental methodologies, independent validation of findings, robust *in vivo* investigations, comprehensive pharmacokinetic and toxicological studies, and well-designed randomized clinical trials will be essential to strengthen the evidence base and facilitate clinical translation. Collectively, these advances have the potential to accelerate the development of safe, effective, and multitargeted anticancer therapeutics derived from underexplored aromatic plants while establishing a stronger scientific foundation for natural product-based precision oncology.

## 4 Conclusion

This systematic review demonstrates that underexplored aromatic plants constitute a valuable reservoir of structurally diverse phytochemicals with promising anticancer potential in preclinical studies. These bioactive compounds exert multitargeted effects by inducing apoptosis, promoting cell-cycle arrest, modulating oxidative stress, inhibiting angiogenesis and metastasis, and regulating key oncogenic signaling pathways. Several phytochemicals emerged as promising lead candidates for anticancer drug discovery; however, their clinical translation remains limited by methodological heterogeneity, inadequate standardization of plant materials and extracts, and insufficient pharmacokinetic, toxicological, and clinical evidence. Importantly, the current evidence is derived predominantly from *in vitro* and *in vivo* studies, and there is no robust evidence from high-quality clinical trials to support the use of aromatic plant-derived compounds or essential oils as established cancer therapies. Therefore, these findings should be regarded as a strong foundation for future drug discovery rather than evidence of proven clinical efficacy.

Future research should prioritize the systematic investigation of underexplored aromatic plant biodiversity using integrated approaches, including metabolomics, multi-omics, artificial intelligence, network pharmacology, and advanced drug-delivery systems. Standardized experimental protocols, rigorous preclinical validation, and well-designed clinical studies will be essential to accelerate the translation of these promising natural products into safe and effective anticancer therapeutics.

## Data Availability

The raw data supporting the conclusions of this article will be made available by the authors, without undue reservation.

## References

[1] Sung H Ferlay J Siegel RL Laversanne M Soerjomataram I Jemal A . Global cancer statistics 2020: GLOBOCAN estimates of incidence and mortality worldwide for 36 cancers in 185 countries. CA: A Cancer J For Clin. (2021) 71:209–49. doi: 10.3322/caac.21660 33538338

[2] Bray F Laversanne M Sung H Ferlay J Siegel RL Soerjomataram I . Global cancer statistics 2022: GLOBOCAN estimates of incidence and mortality worldwide for 36 cancers in 185 countries. CA: A Cancer J For Clin. (2024) 74:229–63. doi: 10.3322/caac.21834 38572751

[3] Dagogo-Jack I Shaw AT . Tumour heterogeneity and resistance to cancer therapies. Nat Rev Clin Oncol. (2018) 15:81–94. doi: 10.1038/nrclinonc.2017.166 29115304

[4] Vasan N Baselga J Hyman DM . A view on drug resistance in cancer. Nature. (2019) 575:299–309. doi: 10.1038/s41586-019-1730-1 31723286 PMC8008476

[5] Newman DJ Cragg GM . Natural products as sources of new drugs over the nearly four decades from 01/1981 to 09/2019. J Nat Prod. (2020) 83:770–803. doi: 10.1021/acs.jnatprod.9b01285 32162523

[6] Atanasov AG Zotchev SB Dirsch VM Supuran CT . Natural products in drug discovery: advances and opportunities. Nat Rev Drug Discov. (2021) 20:200–16. doi: 10.1038/s41573-020-00114-z 33510482 PMC7841765

[7] De Sousa DP Damasceno RO Amorati R Elshabrawy HA De Castro RD Bezerra DP . Essential oils: Chemistry and pharmacological activities. Biomolecules. (2023) 13:1144. doi: 10.3390/biom13071144 37509180 PMC10377445

[8] Siddiqui T Khan MU Sharma V Gupta K . Terpenoids in essential oils: Chemistry, classification, and potential impact on human health and industry. Phytomed Plus. (2024) 4:100549. doi: 10.1016/j.phyplu.2024.100549 38826717

[9] Sharifi-Rad J Sureda A Tenore GC Daglia M Sharifi-Rad M Valussi M . Biological activities of essential oils: From plant chemoecology to traditional healing systems. Molecules. (2017) 22:70. doi: 10.3390/molecules22010070 28045446 PMC6155610

[10] Salehi F Jamali T Kavoosi G Ardestani SK Vahdati SN . Stabilization of Zataria essential oil with pectin-based nanoemulsion for enhanced cytotoxicity in monolayer and spheroid drug-resistant breast cancer cell cultures and deciphering its binding mode with gDNA. Int J Biol Macromol. (2020) 164:3645–55. doi: 10.1016/j.ijbiomac.2020.08.084 32795576

[11] Hanahan D . Hallmarks of cancer: new dimensions. Cancer Discov. (2022) 12:31–46. doi: 10.1158/2159-8290.cd-21-1059 35022204

[12] Wang G Wang JJ Du L Fei L To SS . Inhibitory kinetics and mechanism of flavonoids extracted from Cotinus coggygria Scop. against glioblastoma cancer. Nutr Cancer. (2016) 68:1357–68. doi: 10.1080/01635581.2016.1225105 27673410

[13] Kubatka P Uramova S Kello M Kajo K Samec M Jasek K . Anticancer activities of Thymus vulgaris L. in experimental breast carcinoma *in vitro* and *in vitro*. Int J Mol Sci. (2019) 20:1749. doi: 10.3390/ijms20071749 30970626 PMC6479806

[14] Kubatka P Kello M Kajo K Samec M Jasek K Vybohova D . Chemopreventive and therapeutic efficacy of Cinnamomum zeylanicum L. bark in experimental breast carcinoma: mechanistic *in vitro* and *in vitro* analyses. Molecules. (2020) 25:1399. doi: 10.3390/molecules25061399 32204409 PMC7144360

[15] Alotaibi NM Alotaibi MO Alshammari N Adnan M Patel M . Network pharmacology combined with molecular docking, molecular dynamics, and *in vitro* experimental validation reveals the therapeutic potential of Thymus vulgaris L. essential oil (thyme oil) against human breast cancer. ACS Omega. (2023) 8:48344–59. doi: 10.1021/acsomega.3c07782 38144096 PMC10734022

[16] Bardaweel SK Bakchiche B ALSalamat HA Rezzoug M Gherib A Flamini G . Chemical composition, antioxidant, antimicrobial and antiproliferative activities of essential oil of Mentha spicata L. (Lamiaceae) from Algerian Saharan atlas. BMC Complementary Altern Med. (2018) 18:201. doi: 10.1186/s12906-018-2274-x 29970065 PMC6029017

[17] Mesmar J Abdallah R Hamade K Baydoun S Al-Thani N Shaito A . Ethanolic extract of Origanum Syriacum L. leaves exhibits potent anti-breast cancer potential and robust antioxidant properties. Front Pharmacol. (2022) 13:994025. doi: 10.3389/fphar.2022.994025 36299882 PMC9589507

[18] Diniz do Nascimento L Barbosa de Moraes AA Santana da Costa K Pereira Galúcio JM Taube PS Leal Costa CM . Bioactive natural compounds and antioxidant activity of essential oils from spice plants: New findings and potential applications. Biomolecules. (2020) 10:988. doi: 10.3390/biom10070988 32630297 PMC7407208

[19] Gulati P Chandel A Kumar P Kumar D Kaur D . Medicinal potential of tropical rainforest plants in cancer therapy: Challenges and innovations. Clin Cancer Drugs. (2025) 11:E2212697X402539. doi: 10.2174/012212697x402539251001062221

[20] Karousa MM Kalath H Karam L Suleman M Ayoub MM Fathima A . Chrysopogon zizanioides (Vetiver) essential oil from Qatar targets AKT1 and STAT3 in colorectal and lung cancer: GC-MS profiling, *In vitro* antiproliferative activity, and in silico analyses. Plants. (2026) 15:784. doi: 10.3390/plants15050784 41829818 PMC12986586

[21] Ray A Gadnayak A Jena S Sahoo A Patnaik J Panda PC . Hedychium spicatum rhizome essential oil induces apoptosis in human prostate adenocarcinoma PC-3 cells via mitochondrial stress and caspase activation. Heliyon. (2023) 9:e13807. doi: 10.1016/j.heliyon.2023.e13807 36873474 PMC9981923

[22] Fauquet J Palmieri C Wells M Duez P Blankert B Nachtergael A . Systematic fractionation using Complementary Developing Solvent technique in flash chromatography: A key step in dereplication strategies for natural products. Planta Med. (2026) 92:e70376. doi: 10.1055/s-0045-1815304 41693126

[23] Mbaveng AT Fotso GW Ngnintedo D Kuete V Ngadjui BT Keumedjio F . Cytotoxicity of epunctanone and four other phytochemicals isolated from the medicinal plants Garcinia epunctata and Ptycholobium contortum towards multi-factorial drug resistant cancer cells. Phytomedicine. (2018) 48:112–9. doi: 10.1016/j.phymed.2017.12.016 30195869

[24] Qi SZ Zhang XX Jin Y Wang M Long LP Jing WH . Phenylpropanoid-conjugated pentacyclic triterpenoids from the whole plants of Leptopus lolonum induced cell apoptosis via MAPK and Akt pathways in human hepatocellular carcinoma cells. Bioorg Chem. (2021) 111:104886. doi: 10.1016/j.bioorg.2021.104886 33836342

[25] Nguyen HM Nguyen HT Seephan S Do HB Nguyen HT Ho DV . Antitumor activities of Aspiletrein A, a steroidal saponin from Aspidistra letreae, on non-small cell lung cancer cells. BMC Complementary Med Ther. (2021) 21:87. doi: 10.1186/s12906-021-03262-w 33750378 PMC7941985

[26] Park KR Lee H Kim SH Yun HM . Paeoniflorigenone regulates apoptosis, autophagy, and necroptosis to induce anti-cancer bioactivities in human head and neck squamous cell carcinomas. J Ethnopharmacol. (2022) 288:115000. doi: 10.1016/j.jep.2022.115000 35051602

[27] Zheng YD Bai G Tang C Ke CQ Yao S Tong LJ . 7α, 8α-Epoxynagilactones and their glucosides from the twigs of Podocarpus nagi: Isolation, structures, and cytotoxic activities. Fitoterapia. (2018) 125:174–83. doi: 10.1016/j.fitote.2018.01.007 29355751

[28] Saleh AM Al-Qudah MA Nasr A Rizvi SA Borai A Daghistani M . Comprehensive analysis of the chemical composition and *in vitro* cytotoxic mechanisms of Pallines spinosa flower and leaf essential oils against breast cancer cells. Cell Physiol Biochem. (2017) 42:2043–65. doi: 10.1159/000479900 28803233

[29] Al-Maharik N Salama Y Al-Hajj N Jaradat N Jobran NT Warad I . Chemical composition, anticancer, antimicrobial activity of Aloysia citriodora Palau essential oils from four different locations in Palestine. BMC Complementary Med Ther. (2024) 24:94. doi: 10.1186/s12906-024-04390-9 38365676 PMC10870676

[30] Satapathy SS Bhuyan R Pradhan AK Satpathy S Panda NR Bhuyan SK . Comparative evaluation of anticancer potential of Spilanthes paniculata leaf and flower essential oil using annexin V and orosphere formation in oral cancer cell line. Med Oncol. (2024) 41:259. doi: 10.1007/s12032-024-02523-1 39369172

[31] Zhang J Chen T Gao Y . Tumor-suppressing efficacy of essential oil of Boswellia serrata gum resin and its synergistic effect on doxorubicin-induced growth inhibition of breast cancer cells. 3 Biotech. (2026) 16:147. doi: 10.1007/s13205-026-04759-2 41853213 PMC12992863

[32] Parums DV . Review articles, systematic reviews, meta-analysis, and the updated preferred reporting items for systematic reviews and meta-analyses (PRISMA) 2020 guidelines. Med Sci Monitor: Int Med J Exp Clin Res. (2021) 27:e934475-1. doi: 10.12659/msm.934475 34421116 PMC8394590

[33] Abdalla MS Salem AA Hussien ET Ramadan SS . Broad-spectrum bioactivities and therapeutic potential of essential oils of certain aromatic and medicinal plants and their combination. Sci Rep. (2025) 15:32463. doi: 10.1038/s41598-025-18314-1 40940369 PMC12432102

[34] Aindelis G Spyridopoulou K Kyriakou S Tiptiri-Kourpeti A Panayiotidis MI Pappa A . Evaluating the chemical composition and antitumor activity of Origanum vulgare ssp. hirtum essential oil in a preclinical colon cancer model. Int J Mol Sci. (2025) 26:4737. doi: 10.3390/ijms26104737 40429884 PMC12111866

[35] Almnhawy M Jebur M Alhajamee M Marai K Tabrizi MH . PLGA-based nano-encapsulation of Trachyspermum ammi seed essential oil (TSEO-PNP) as a safe, natural, efficient, anticancer compound in human HT-29 colon cancer cell line. Nutr Cancer. (2021) 73:2808–20. doi: 10.1080/01635581.2020.1862256 33319599

[36] Baraich A Elbouzidi A Haddou M Taibi M Sadougui I Bayad EM . Phytochemical and pharmacological elucidation of Jasonia glutinosa (L.) fourr. essential oil: a promising therapeutic source against inflammation and cancer. Med Oncol. (2026) 43:149. doi: 10.1007/s12032-026-03245-2 41670759

[37] Chang CT Soo WN Chen YH Shyur LF . Essential oil of Mentha aquatica var. Kenting water mint suppresses two-stage skin carcinogenesis accelerated by BRAF inhibitor vemurafenib. Molecules. (2019) 24:2344. doi: 10.3390/molecules24122344 31242703 PMC6630265

[38] Chen GR Chang ML Chang ST Ho YT Chang HT . Cytotoxicity and apoptosis induction of 6, 7-dehydroroyleanone from Taiwania cryptomerioides bark essential oil in hepatocellular carcinoma cells. Pharmaceutics. (2022) 14:351. doi: 10.3390/pharmaceutics14020351 35214084 PMC8880271

[39] Lima EJ Alves RG D´ Elia GM Anunciação TA Silva VR Santos LD . Antitumor effect of the essential oil from the leaves of Croton matourensis Aubl.(Euphorbiaceae). Molecules. (2018) 23:2974. doi: 10.3390/molecules23112974 30441836 PMC6278459

[40] Deng K Wei Y Yu K Mei Y Han S Yan Q . Revealing the antitumor mechanisms of aromatic oil from Amomum villosum through integrated network pharmacology, bioinformatics, machine learning, single-cell sequencing, and cell experiments. Biochem Biophys Res Commun. (2026) 796:153153. doi: 10.1016/j.bbrc.2025.153153 41421224

[41] Dervişoğlu G . Chemical profiling, molecular docking, and mechanistic anticancer activity of Pinus sylvestris essential oil in SH-SY5Y and U-87MG cells. Molecules. (2026) 31:470. doi: 10.3390/molecules31030470 41683447 PMC12898508

[42] Dolghi A Coricovac D Dinu S Pinzaru I Dehelean CA Grosu C . Chemical and antimicrobial characterization of Mentha piperita L. and Rosmarinus officinalis L. essential oils and *in vitro* potential cytotoxic effect in human colorectal carcinoma cells. Molecules. (2022) 27:6106. doi: 10.3390/molecules27186106 36144839 PMC9505364

[43] Ravi Y Vethamoni PI Saxena SN Kaviyapriya M Santhanakrishnan VP Raveendran M . Anticancer potential of Thymoquinone from Nigella sativa L.: an in-silico and cytotoxicity study. PloS One. (2025) 20:e0323804. doi: 10.1371/journal.pone.0323804 40504815 PMC12161580

[44] Raza W Meena A Luqman S . Antitumor effects of diosmetin in Ehrlich ascites carcinoma mouse model: insights into ROS-mediated mechanisms. Naunyn-Schmiedeberg's Arch Pharmacol. (2025) 399:5663–75. doi: 10.1007/s00210-025-04617-7 41212275

[45] Kumar S Pandey AR Singh SP Swain A Bisen AC Agrawal S . Synthesis and evaluation of lactam and maleimide derivatives of 16-hydroxycleroda-3, 13 (14) Z-dien-15, 16-olide from Polyalthia longifolia as potential anticancer agents. Eur J Med Chem. (2025) 297:117894. doi: 10.1016/j.ejmech.2025.117894 40602225

[46] Mothana RA Khaled JM Noman OM Kumar A Alajmi MF Al-Rehaily AJ . Phytochemical analysis and evaluation of the cytotoxic, antimicrobial and antioxidant activities of essential oils from three Plectranthus species grown in Saudi Arabia. BMC Complementary Altern Med. (2018) 18:237. doi: 10.1186/s12906-018-2302-x 30097042 PMC6086039

[47] Mothana RA Nasr FA Khaled JM Al-Zharani M Noman OM Abutaha N . Analysis of chemical composition and assessment of cytotoxic, antimicrobial, and antioxidant activities of the essential oil of Meriandra dianthera growing in Saudi Arabia. Molecules. (2019) 24:2647. doi: 10.3390/molecules24142647 31336582 PMC6680587

[48] Saraiva N Costa JG Reis C Almeida N Rijo P Fernandes AS . Anti-migratory and pro-apoptotic properties of parvifloron D on triple-negative breast cancer cells. Biomolecules. (2020) 10:158. doi: 10.3390/biom10010158 31963771 PMC7023143

[49] Saeedi F Salehi M Kamali MJ Mir MA Kazemi S Shirafkan F . Evaluation of the cytotoxic activities of the essential oil from Pistacia atlantica Desf. oleoresin on human gastric cancer cell lines. Med Oncol. (2024) 41:148. doi: 10.1007/s12032-024-02339-z 38733486

[50] Saini D Chaudhary PK Chaudhary JK Kaur H Verma GK Pramanik SD . Molecular mechanisms of antiproliferative and pro-apoptotic effects of essential oil active constituents in MCF7 and T24 cancer cell lines: *In vitro* insights and in silico modelling of proapoptotic gene product-compound interactions. Apoptosis. (2025) 30:805–25. doi: 10.1007/s10495-024-02065-x 39738801

[51] Wu JG Peng W Yi J Wu YB Chen TQ Wong KH . Chemical composition, antimicrobial activity against Staphylococcus aureus and a pro-apoptotic effect in SGC-7901 of the essential oil from Toona sinensis (A. Juss.) Roem. leaves. J Ethnopharmacol. (2014) 154:198–205. doi: 10.1016/j.jep.2014.04.002 24726685 PMC7126815

[52] Yang BJ Wang J Zeng ZQ Yang X Huang AY Hao XJ . Sesquiterpene lactones from Carpesium abrotanoides L. and their activity in inducing protective autophagy. Nat Prod Res. (2022) 36:3207–10. doi: 10.1080/14786419.2021.1955881 34498970

[53] Živković M Stanisavljević I Gajović N Pavlović S Simović Marković B Jovanović IP . Comprehensive phytochemical analysis and evaluation of antioxidant, antimicrobial, cytotoxic, and immunomodulatory activities of commercial cinnamon bark essential oil (Cinnamomum zeylanicum L.). Int J Mol Sci. (2025) 26:6482. doi: 10.3390/ijms26136482 40650258 PMC12250438

[54] Semiz G Mutlu D Günal B Semiz A Arslan Ş . The anticancer effect of Salvia pisidica essential oil through promotion intrinsic and extrinsic apoptosis pathways in human cancer cell lines. J Herb Med. (2023) 39:100664. doi: 10.1016/j.hermed.2023.100664 38826717

[55] Sheashea AA Ahmed FA El Zayat E Ebeed BW Elberry MH Hassan ZK . *In vitro* antiviral and anticancer effects of Tanacetum sinaicum essential oil on human cervical and breast cancer. Asian Pacific J Cancer Prevention: APJCP. (2024) 25:1457. doi: 10.31557/APJCP.2024.25.4.1457 PMC11162737 38680008

[56] Singh B Prajapati KS Kumar A Patel S Kumar S Jaitak V . Chemical composition, *In vitro* and in silico evaluation of essential oil from Ocimum tenuiflorum and Coriandrum sativum Linn for lung cancer. Curr Comput-Aided Drug Des. (2024) 20:628–39. doi: 10.2174/1573409920666230831144716 37653637

[57] Spyridopoulou K Fitsiou E Bouloukosta E Tiptiri-Kourpeti A Vamvakias M Oreopoulou A . Extraction, chemical composition, and anticancer potential of Origanum onites L. essential oil. Molecules. (2019) 24:2612. doi: 10.3390/molecules24142612 31323754 PMC6680447

[58] Taherkhani M . *In vitro* cytobiological effects of phytochemicals from Artemisia turanica. J Rep Pharm Sci. (2019) 8:106–13. doi: 10.4103/jrptps.jrptps_37_18

[59] Tesfaye S Birkel L Bandaru SS Barrera-Adame DA Eichelbaum M Lulekal E . Bioassay-guided isolation and identification of antiproliferative compounds from Acokanthera schimperi (A. DC.) Schweinf and Gnidia involucrata Steudel ex A. rich. Fitoterapia. (2026) 189:107090. doi: 10.1016/j.fitote.2026.107090 41534672

[60] Vaglica A Maggio A Badalamenti N Bruno M Lauricella M D'Anneo A . Seseli bocconei Guss. and S. tortuosum subsp. maritimum Guss. essential oils inhibit colon cancer cell viability. Fitoterapia. (2023) 170:105672. doi: 10.1016/j.fitote.2023.105672 37709102

[61] Reyes-Vidal I Tepale-Ledo I Rivera G Ortiz-Islas E Pérez-Mora S Pérez-Ishiwara DG . In silico and *In vitro* evaluation of δ-cadinene from Decatropis bicolor as a selective inhibitor of human cell adhesion and invasion proteins. Cancers. (2025) 17:2839. doi: 10.3390/cancers17172839 40940940 PMC12427431

[62] Harassi Y Tilaoui M Idir A Frédéric J Baudino S Ajouaoi S . Phytochemical analysis, cytotoxic and antioxidant activities of Myrtus communis essential oil from Morocco. J Complementary Integr Med. (2019) 16:20180100. doi: 10.1515/jcim-2018-0100 30661057

[63] Muhasina MK Uddin ME Mundavath RN Swaroop AK Nilewar SS Mariappan E . Developing a formulation of Tinospora cordifolia, identifying its active components, and assessing its anticancer effects on breast cancer cell lines. Med Oncol. (2025) 42:312. doi: 10.1007/s12032-025-02777-3 40617988

[64] Kadyrbay A Ibragimova LN Iwan M Ludwiczuk A Biernasiuk A Sakipova ZB . Essential oil from the aerial parts of Artemisia serotina Bunge (Winter Wormwood) growing in Kazakhstan-phytochemical profile and bioactivity. Molecules. (2025) 30:2956. doi: 10.3390/molecules30142956 40733222 PMC12297878

[65] Ferreira OO Costa VG Cruz JN Kataoka MS Pinheiro JD Nascimento LD . Chemical composition and *in vitro* and in silico biological activities of Myrciaria tenella (DC.) O. Berg (Myrtaceae) essential oil from Brazil. J Food Biochem. (2024) 2024:2848736. doi: 10.1155/2024/2848736

[66] Ghavam M . Heracleum persicum Desf. ex Fisch. CA Mey. & Avé-Lall. fruit essential oil: content, antimicrobial activity and cytotoxicity against ovarian cancer cell line. BMC Complementary Med Ther. (2023) 23:87. doi: 10.1186/s12906-023-03892-2 36944973 PMC10029216

[67] Estanislao Gómez CC Aquino Carreño A Pérez Ishiwara DG San Martín Martínez E Morales López J Pérez Hernández N . Decatropis bicolor (Zucc.) Radlk essential oil induces apoptosis of the MDA-MB-231 breast cancer cell line. BMC Complementary Altern Med. (2016) 16:266. doi: 10.1186/s12906-016-1136-7 27491777 PMC4974778

[68] Gruin S Creţu O Mioc A Mioc M Prodea A Atyim E . Evaluation of Cedrus atlantica essential oil: chemical composition, anticancer activity and molecular docking studies. Molecules. (2025) 31:46. doi: 10.3390/molecules31010046 41515343 PMC12786621

[69] Guesmi F Tahri W Mehrez A Barkaoui T Prasad S Giuffrè AM . Colorectal carcinoma cell targeting aromatherapy with Teucrium ramosissimum essential oil to sensitize TRAIL/Apo2L-induced HCT-116 cell death. Int Immunopharmacol. (2024) 136:112405. doi: 10.1016/j.intimp.2024.112405 38850792

[70] El Hajj J Karam L Jaber A Cheble E Akoury E Kobeissy PH . Evaluation of antiproliferative potentials associated with the volatile compounds of Lantana camara flowers: selective *in vitro* activity. Molecules. (2024) 29:5431. doi: 10.3390/molecules29225431 39598820 PMC11597665

[71] Pant P Sut S Castagliuolo I Gandin V Maggi F Gyawali R . Sesquiterpene rich essential oil from Nepalese Bael tree (Aegle marmelos (L.) Correa) as potential antiproliferative agent. Fitoterapia. (2019) 138:104266. doi: 10.1016/j.fitote.2019.104266 31302251

[72] Pheko-Ofitlhile T Makhzoum A . Impact of hydrodistillation and steam distillation on the yield and chemical composition of essential oils and their comparison with modern isolation techniques. J Essent Oil Res. (2024) 36:105–15. doi: 10.1080/10412905.2024.2320350 37339054

[73] Ali SS Al-Tohamy R Al-Zahrani M Badr A Sun J . Essential oils and plant-derived bioactive compounds: a comprehensive review of their therapeutic potential, mechanisms of action, and advances in extraction technologies. Phytochem Rev. (2026) 25:223–71. doi: 10.1007/s11101-025-10123-8 30311153

[74] Ojong C Besong SA Aryee AN . Solvent-based extraction recovers phytochemicals from medicinal plants demonstrating anticancer and chemopreventive potential: a review. Molecules. (2026) 31:1202. doi: 10.3390/molecules31071202 41976242 PMC13075112

[75] Mani J Johnson J Hosking H Hoyos BE Walsh KB Neilsen P . Bioassay guided fractionation protocol for determining novel active compounds in selected Australian flora. Plants. (2022) 11:2886. doi: 10.3390/plants11212886 36365337 PMC9654191

[76] Kour J Lone BA Kumar A Ganai BA Yadav G Gupta P . Bioassay-guided isolation and identification of antimutagenic compounds from Morina coulteriana and evaluation of its therapeutic potential. Phytomed Plus. (2025) 5:100676. doi: 10.1016/j.phyplu.2024.100676 38826717

[77] Arunachalam S Nagoor Meeran MF Azimullah S Jha NK Saraswathiamma D Subramanya S . α-Bisabolol attenuates doxorubicin induced renal toxicity by modulating NF-κB/MAPK signaling and caspase-dependent apoptosis in rats. Int J Mol Sci. (2022) 23:10528. doi: 10.3390/ijms231810528 36142441 PMC9502245

