# Supplementary material for: Uncharted phytochemical frontiers: a systematic review of novel bioactive compounds from underexplored aromatic plants for cancer therapy
Source: Front Oncol. 2026 Aug 28;16:1924878. doi: 10.3389/fonc.2026.1924878 (PMC13561809; doi:10.3389/fonc.2026.1924878)
Supplement: Supplementary file 1 [file Table1.docx]

Table S1: Details of the systematic literature search strategy.

| **Database** | **Search Date** | **Search Period** | **Search Strategy (exact search string)** | **Filters Applied** | **Records Retrieved** |
| --- | --- | --- | --- | --- | --- |
| PubMed | 31 March 2026 | Jan 2000-Mar 2026 | (("Plants, Medicinal"[Mesh] OR "Aromatic Plants"[tiab] OR "medicinal plant*"[tiab] OR "aromatic plant*"[tiab] OR "underexplored plant*"[tiab]) AND (Phytochemicals [Mesh] OR "Plant Extracts"[Mesh] OR phytochemical*[tiab] OR "bioactive compound*"[tiab] OR extract*[tiab] OR fraction*[tiab]) AND (Neoplasms [Mesh] OR cancer*[tiab] OR anticancer*[tiab] OR antitumor*[tiab]) AND ("Pharmacological Actions"[Mesh] OR bioactiv*[tiab] OR cytotoxic*[tiab] OR therapeutic*[tiab])) | English; Original articles | 2699 |
| Scopus | 31 March 2026 | Jan 2000-Mar 2026 | ((INDEXTERMS("Plants, Medicinal") OR TITLE-ABS("Aromatic Plants") OR TITLE-ABS("medicinal plant*") OR TITLE-ABS("aromatic plant*") OR TITLE-ABS("underexplored plant*")) AND (INDEXTERMS(Phytochemicals) OR INDEXTERMS("Plant Extracts") OR TITLE-ABS(phytochemical*) OR TITLE-ABS("bioactive compound*") OR TITLE-ABS(extract*) OR TITLE-ABS(fraction*)) AND (INDEXTERMS(Neoplasms) OR TITLE-ABS(cancer*) OR TITLE-ABS(anticancer*) OR TITLE-ABS(antitumor*)) AND (INDEXTERMS("Pharmacological Actions") OR TITLE-ABS(bioactiv*) OR TITLE-ABS(cytotoxic*) OR TITLE-ABS(therapeutic*))) | English; Article | 2515 |
| ScienceDirect (Embase) | 31 March 2026 | Jan 2000-Mar 2026 | (('Plants, Medicinal'/exp OR 'Aromatic Plants':ti,ab OR 'medicinal plant*':ti,ab OR 'aromatic plant*':ti,ab OR 'underexplored plant*':ti,ab) AND (Phytochemicals/exp OR 'Plant Extracts'/exp OR phytochemical*:ti,ab OR 'bioactive compound*':ti,ab OR extract*:ti,ab OR fraction*:ti,ab) AND (Neoplasms/exp OR cancer*:ti,ab OR anticancer*:ti,ab OR antitumor*:ti,ab) AND ('Pharmacological Actions'/exp OR bioactiv*:ti,ab OR cytotoxic*:ti,ab OR therapeutic*:ti,ab)) | Research articles | 8426 |
